# Dose adjustment of follicle-stimulating hormone (FSH) during ovarian stimulation as part of medically-assisted reproduction in clinical studies: a systematic review covering 10 years (2007–2017)

**DOI:** 10.1186/s12958-021-00744-x

**Published:** 2021-05-11

**Authors:** Human Fatemi, Wilma Bilger, Deborah Denis, Georg Griesinger, Antonio La Marca, Salvatore Longobardi, Mary Mahony, Xiaoyan Yin, Thomas D’Hooghe

**Affiliations:** 1 ART Fertility Clinics, Abu Dhabi & Dubai and Muscat Royal Marina Village, Abu Dhabi, United Arab Emirates; 2 Medical Affairs Fertility, Endocrinology & General Medicine, Merck Serono GmbH (an affiliate of Merck KGaA, Darmstadt, Germany), Darmstadt, Germany; 3 Global Clinical Development, EMD Serono Research and Development Institute, Inc (an affiliate of Merck KGaA, Darmstadt, Germany), Billerica, MA USA; 4 Department of Gynecological Endocrinology and Reproductive Medicine, University Hospital Schleswig-Holstein, Lübeck, Germany; 5 Dipartimento di Scienze Mediche e Chirurgiche Materno-Infantili e dell’Adulto, University of Modena and Reggio Emilia and Clinica Eugin Modena, Modena, Italy; 6 Global Clinical Development, Merck Serono S.p.A (an affiliate of Merck KGaA, Darmstadt, Germany), 00176 Rome, Italy; 7 Medical Affairs - Endocrinology/Reproductive Health, EMD Serono, Inc (an affiliate of Merck KGaA, Darmstadt, Germany), Rockland, MA USA; 8 Research & Development, EMD Serono, Inc (an affiliate of Merck KGaA, Darmstadt, Germany), Billerica, MA USA; 9 Global Medical Affairs Fertility, Merck KGaA, Darmstadt, Germany; 10 Department of Development & Regeneration, University of Leuven (KU Leuven), Leuven, Belgium; 11 Department of Obstetrics Gynecology, Yale University, New Haven, CT USA

**Keywords:** Follicle-stimulating hormone (FSH), FSH dose adjustment, Recombinant-human FSH, Follitropin, Controlled ovarian stimulation (COS), In vitro fertilization (IVF), Medically-assisted reproduction, Assisted reproductive technology (ART) treatment, Systematic review

## Abstract

**Background:**

Individualization of the follicle-stimulating hormone (FSH) starting dose is considered standard clinical practice during controlled ovarian stimulation (COS) in patients undergoing assisted reproductive technology (ART) treatment. Furthermore, the gonadotropin dose is regularly adjusted during COS to avoid hyper- or hypo-ovarian response, but limited data are currently available to characterize such adjustments. This review describes the frequency and direction (increase/decrease) of recombinant-human FSH (r-hFSH) dose adjustment reported in clinical trials.

**Methods:**

We evaluated the proportion of patients undergoing ART treatment who received ≥ 1 r-hFSH dose adjustments. The inclusion criteria included studies (published Sept 2007 to Sept 2017) in women receiving ART treatment that allowed dose adjustment within the study protocol and that reported ≥ 1 dose adjustments of r-hFSH; studies not allowing/reporting dose adjustment were excluded. Data on study design, dose adjustment and patient characteristics were extracted. Point-incidence estimates were calculated per study and overall based on pooled number of cycles with dose adjustment across studies. The Clopper–Pearson method was used to calculate 95% confidence intervals (CI) for incidence where adjustment occurred in < 10% of patients; otherwise, a normal approximation method was used.

**Results:**

Initially, 1409 publications were identified, of which 318 were excluded during initial screening and 1073 were excluded after full text review for not meeting the inclusion criteria. Eighteen studies (6630 cycles) reported dose adjustment: 5/18 studies (1359 cycles) reported data for an unspecified dose adjustment (direction not defined), in 10/18 studies (3952 cycles) dose increases were reported, and in 11/18 studies (5123 cycles) dose decreases were reported. The studies were performed in women with poor, normal and high response, with one study reporting in oocyte donors and one in obese women. The median day that dose adjustment was permitted was Day 6 after the start of treatment. The point estimates for incidence (95% CI) for unspecified dose adjustment, dose increases, and dose decreases were 45.3% (42.7, 48.0), 19.2% (18.0, 20.5), and 9.5% (8.7, 10.3), respectively.

**Conclusions:**

This systematic review highlights that, in studies in which dose adjustment was allowed and reported, the estimated incidence of r-hFSH dose adjustments during ovarian stimulation was up to 45%.

**Supplementary Information:**

The online version contains supplementary material available at 10.1186/s12958-021-00744-x.

## Background

There are many aspects of assisted reproductive technology (ART) treatment that can be individualized to optimize treatment outcomes, including controlled ovarian stimulation (COS), ovulation triggering and luteal phase support [1–3]. As with all medically-assisted reproduction treatments, the approach to patient care should be individualized according to the characteristics of each patient and monitored to ensure that the response is optimized with respect to efficacy and safety [4]. Evidence from routine care settings and randomized trials in which the individualized approach to reproductive medicine differs from a standardized approach may provide targeted evidence regarding the advantages or disadvantages of an individualized approach.

Individualization during COS can include the selection of down-regulation protocols (e.g. gonadotropin-releasing hormone [GnRH] agonist or antagonist), gonadotropin type, gonadotropin starting dose (with 100–225 IU generally considered the standard gonadotropin daily dose [5, 6]), gonadotropin dose adjustment during ovarian stimulation, type of trigger of final oocyte maturation, and type and duration of luteal phase support [7–9]. It is important to distinguish that individualization of the gonadotropin dose may be implemented at two different time points: at the start of each new treatment cycle (‘starting dose selection’), or during the course of ovarian stimulation within a given cycle (‘dose adjustment during treatment/cycle’) [8, 10–13], even though it is unclear if this individualized approach is beneficial in terms of live birth rate according to the ESHRE guidelines for ovarian stimulation during ART treatment [14]. Likewise, dose individualization in patients with expected and unexpected high ovarian response may decrease the risk of ovarian hyperstimulation syndrome (OHSS) and cycle cancellation.

### Starting dose selection

Starting dose selection is usually based on patient characteristics and established biomarkers for treatment response, including AMH and AFC, Day 3 follicle-stimulating hormone (FSH), age, body weight, response to any previous ovarian stimulation cycle (ovulation induction [OI] gonadotropin and/or ART treatment) and any specific diagnoses relating to subfertility (including polycystic ovary syndrome [PCOS], amenorrhea, thyroid stimulating hormone levels and conditions of the Fallopian tubes) [14–19]. Tailoring the FSH starting dose to the individual patient is considered to be standard clinical practice by some healthcare professionals, and a nomogram has been developed based on age, AMH and AFC to help clinicians calculate daily FSH starting doses [18].

Patients with reduced ovarian response after ovarian stimulation for ART can be categorized according to the POSEIDON criteria, based on age and expected euploidy rate, ovarian biomarkers (antral follicle count [AFC] and anti-Müllerian hormone [AMH]), and ovarian response if a previous stimulation has been performed [20]. Individualizing the gonadotropin dose according to whether a patient has expected low ovarian response (POSEIDON Groups 3 and 4; AFC < 5, AMH < 1.2 ng/ml) or unexpected low ovarian response (POSEIDON Groups 1 and 2; AFC ≥ 5, AMH ≥ 1.2 ng/ml) may mitigate against the risk of poor ovarian response (POR) and/or cycle cancellation [7–9, 21]. Cycle cancellation and/or the retrieval of no or very few viable oocytes owing to poor ovarian response may necessitate further COS cycles or discontinuation of the in vitro fertilization (IVF) procedure, which increases both the financial and emotional burden on patients [13] as well as time to live birth. However, based on the current evidence, the ESHRE guideline on ovarian stimulation was unclear whether a higher gonadotropin dose (> 150 IU) was recommended for patients with predicted poor response, and a dose higher than 300 IU was not recommended for this patient group [14]. In addition, some studies suggest that higher gonadotropin doses during COS may be detrimental [22–25].

Excessive ovarian response to COS may also result in increased rates of cycle cancellation as well as an increased risk of OHSS [26]. There is significant evidence that, in patients predicted to have a hyper-response to FSH, decreasing the FSH starting dose may reduce the risk of OHSS [26]. In a recent study, women with a predicted hyper-response (AFC > 15) were randomized to an FSH daily dose of 100 IU (*n* = 255) or 150 IU (*n* = 266). There were no relevant differences in live birth rates between the groups, but the lower FSH dose was associated with a reduced incidence of mild and moderate OHSS, with no impact on severe OHSS [27]. Similarly, according to a pooled data analysis from four studies [28], live birth rates were similar in patients receiving adjustment of gonadotropin starting dose and those receiving standard starting dosing (OR 1.04 [95% CI 0.88, 1.23]) but a reduction in the incidence of moderate to severe OHSS (OR 0.58; 95% CI 0.34 to 1.00) was observed, suggesting a safety benefit.

### Dose adjustment during treatment

There is good evidence that practitioners regularly adjust the gonadotropin dose during ovarian stimulation: dose adjustment was reported in up to 41% of cycles according to an analysis of routine clinical practice in the USA [29], probably because clinicians consider that adjusting the dose during COS represents individualized care to improve efficacy and safety during ART treatment.

Dose adjustment during treatment is usually based on ovarian response, measured by ultrasound assessment of follicular development and hormonal monitoring [14], with the aim of avoiding hypo- or hyper-ovarian responses, thereby optimizing outcome and safety and potentially also the endocrine profile, oocyte quality and endometrial receptivity. Doses for the majority of FSH preparations, including follitropin alfa and follitropin beta, can be adjusted at the start as well as during a treatment cycle. For example, for the reference recombinant follitropin alfa, a starting dose of 150–225 IU per day is indicated, with adjustment after 3–5 days of 75–150 IU not to exceed 450 IU/day [30]; and for the reference recombinant follitropin beta, a starting dose of 200 IU per day is recommended, with adjustment after ≥ 7 days of treatment, or after 6 days in patients with high response [31]. However, dose adjustment during treatment of some preparations is restricted by galenic formulation or specific usage instructions [9, 32]. Currently, dose adjustment with 12.5 IU increments are possible in specific formulations for follitropin alpha [33–38]. Furthermore, there is increasing evidence suggesting the benefit of devices that allow small dose changes for reducing the risk of OHSS [39–41], although it needs to be confirmed whether using these devices would lead to improved clinical outcomes.

Dose adjustment may have particular benefits for specific patient groups. To maximize the ovarian potential, reduce the time to live birth and increase cumulative live birth rates, as many oocytes as possible need to be retrieved without putting the patient at risk for OHSS [42–44]. Patients with unexpected low ovarian response (Poseidon Groups 3 and 4) demonstrate an initial slow response to FSH stimulation in terms of estradiol levels and follicle growth [12]; therefore, increasing the FSH dose during treatment in these patients may increase the number of oocytes retrieved. However, trials reporting dose adjustment in poor responders are usually designed to assess individualization of the starting dose based on pretreatment assessment (e.g., AMH or AFC) in patients with expected poor response, making it difficult to assess the benefit of dose adjustment only during treatment in unexpected poor responders.

In patients with an unexpected hyper-response to FSH, decreasing the FSH dose during treatment may reduce the occurrence of OHSS; with moderate-to-severe OHSS estimated to arise in 0.5 to 5.0% of IVF cycles [45, 46]. However, similar to unexpected poor responders, most trials that report dose adjustment in hyper responders are designed to assess individualization of the starting dose in patients with expected hyper response, making it difficult to evaluate dose adjustments during treatment in patients with unexpected hyper-responses.

### Aims

Although dose adjustment was reported in up to 41% of cycles according to an analysis of routine clinical practice in the USA [29], it is not known to what extent gonadotropin dose adjustment occur during the highly controlled research environment of a clinical trial. In some clinical trials, dose adjustment during ovarian stimulation may not be allowed, to reduce the variability of the intervention between patients. In other clinical trials, dose adjustment may be allowed, but the actual incidence of dose adjustment may not always be reported. The aim of this systematic review was to describe the frequency and direction (increase/decrease) of recombinant human (r-h) FSH dose adjustment reported in clinical trials that allowed dose adjustment within the study protocol, involving patients undergoing COS as part of IVF treatment.

## Methods

### Study protocol

The protocol used in this review is described below and has not been registered with any repositories. PubMed was searched to identify publications reporting the proportion of patients undergoing COS for IVF receiving dose adjustment, covering a period of 10 years (6 Sept 2007 to 6 Sept 2017).

### Literature search

The following search string was used: (((“follitropin alfa” OR “follitropin alpha” OR “follitropin beta” OR “follitropin delta” OR “follicle stimulating hormone” OR “follicle-stimulating hormone” OR FSH OR hfsh OR rhfsh OR rfsh OR “h-FSH” OR “rh-FSH” OR “r-hFSH” OR “r-h-FSH” OR “r-FSH” OR “u-FSH” OR ufsh OR “uh-FSH” OR “u-hFSH” OR “u-h-FSH” OR “gonal-f” OR gonal f OR gonal OR “SJ-0021” OR SJ0021 OR bemfola OR folia OR ovaleap OR XM17 OR “XM-17” OR follitropin OR corifollitropin OR rekovelle OR “FE-999049” OR FE999049 OR elonva OR “FSH-CTP” OR “ML-8962” OR ML8962 OR “ORG-36286” OR ORG36286 OR “SCH-900962” OR SCH900962 OR follistim OR puregon OR “ORG-32489” OR ORG32489 OR Bravelle OR “HP-FSH” OR “HP-uFSH” OR “HP-u-FSH” OR “HP-uhFSH” OR “HP-u-hFSH” OR “HP-uh-FSH” OR menotropin))) AND ((“controlled ovarian stimulation” OR COS OR “controlled ovarian hyperstimulation” OR “ovarian stimulation” OR IVF OR “in vitro fertilization” OR “in vitro fertilisation” OR IUI OR “intrauterine insemination” OR “artificial insemination” OR ART OR “assisted reproductive” OR “assisted reproduction” OR “oocyte induction OR OI OR “oocyte retrieval”) OR (Reproductive Techniques [MeSH Terms])).

### Study selection

The search was limited to a 10-year period (in order to reflect recent/current practice and to ensure that the number of articles to be screened was manageable) starting 6 Sept 2007, and filtered to include only studies written in English and conducted in humans. The inclusion criteria used to filter the results were: clinical trials (including prospective and retrospective studies) in women receiving ART treatment that allowed dose adjustment within the study protocol, with ≥ 1 dose adjustments of r-hFSH actually reported. Only studies that used r-hFSH were included in the data analysis (r-hFSH was selected in order to have a defined patient population; furthermore, evaluating all gonadotropins would have resulted in an unmanageable number of articles to screen); any comparator study arms using urinary FSH alone (i.e. without r-hFSH) were excluded. Only papers with primary reporting of results were included, whereby if data from a study were reported multiple times (i.e. secondary reporting), only the first publication reporting dose adjustment was included. The exclusion criteria used to filter the results were: congress abstracts; review articles; reports of studies that did not involve r-hFSH treatment; studies that did not allow gonadotropin dose adjustment by protocol, studies that did allow dose adjustment but where no data on dose adjustment were reported.

Abstracts were initially screened by AJ to remove studies that were explicitly unrelated to the study question or that did not meet the inclusion or exclusion criteria. After initial screening, the full text of remaining articles was obtained and screened by AJ, DD, SL, WB, MM and TDH to identify relevant publications. Any disagreement was resolved by discussion.

### Data collection

Data on study design, dose adjustment and patient characteristics were extracted during screening. The data extraction forms are included in Supplementary Table 1. Dose adjustment frequency data were recorded overall and also according to the direction of dose adjustment (i.e. increases/decreases in dose; or ‘unspecified’ for those studies that did not define the direction of the dose adjustment) according to how they were reported in the identified publications. The risk of bias in individual studies and across studies was not assessed, as this was a scoping review [47] in which the outcome evaluated should not be affected by subjective bias as no analysis or comparison between groups was considered.

### Statistical analysis

Point estimates for incidences were reported per study and overall based on pooled number of cycles with dose adjustment across studies. The Clopper–Pearson method was used to calculate 95% confidence intervals (CI) for incidence where adjustment occurred in < 10% of patients; otherwise, a normal approximation method was used. Data were also analyzed according to the GnRH protocol (agonist vs antagonist).

## Results

### Study selection

**Fig. 1:**
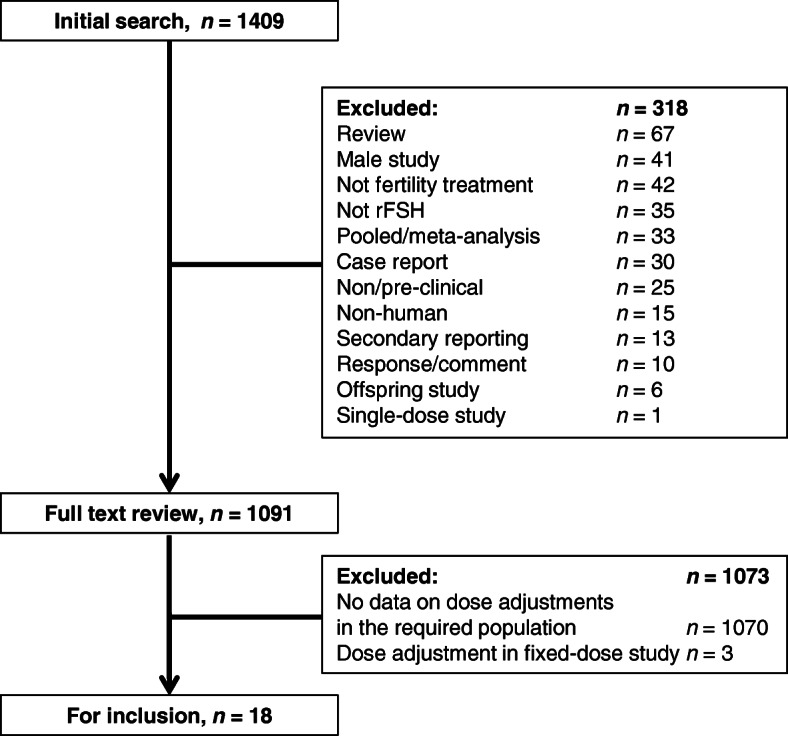
Study screening and selection

A total of 1409 publications were initially identified by the search, of which 318 studies were excluded during initial screening due to the fact they did not meet the inclusion criteria. A further 1073 studies were excluded following full-text review, as either no data on dose adjustment were available in the required population or dose adjustment were reported in fixed dose studies that did not allow dose adjustment in their study design (i.e. where adjusting the dose resulted in a protocol deviation). Eighteen out of the 1409 publications searched reported dose adjustment and were eligible for review [9, 40, 48–63]. Full screening details are shown in Fig. 1.

### Study characteristics

**Fig. 2:**
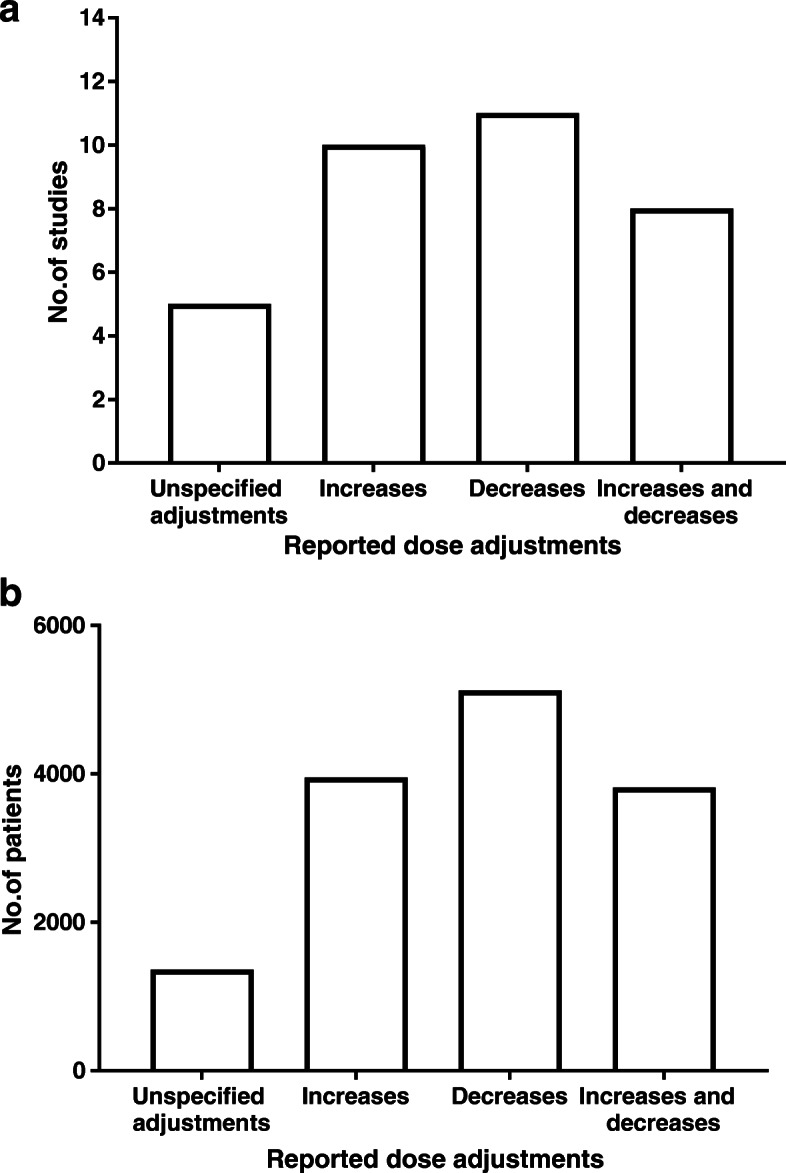
Number of **a** studies and **b** cycles with ≥ 1 dose adjustment of FSH

In the 18 included studies, data were available for 6630 cycles. Five studies (1359 cycles) reported data for unspecified dose adjustments (increases or decreases; direction not defined), 10 studies (3952 cycles) reported dose increases, 11 studies (5123 cycles) reported dose decreases, and eight studies (3813 cycles) reported both dose increases and decreases (Fig. 2). Seven studies (1994 cycles) used GnRH agonists, eight studies (2411 cycles) used GnRH antagonists, and three studies either did not specify or used both agonists and antagonists. Ten studies were randomized controlled trials (RCTs) and three studies were done for marketing authorization purposes [54, 62, 63]. The studies were performed in patients with a predicted poor (*n* = 2), normal (*n* = 3) or high response (*n* = 6) (mean AFC [standard deviation (SD)] ranged from 5.3 [4.29] to 21.6 [12.0]; mean AMH [SD] ranged from 1.7 [2.06] to 27 [20]; Supplementary Table 1), with 7 studies not reporting AFC/AMH data to determine the predicted response. Two studies reported in non-standard populations (1 in oocyte donors [59] and 1 in obese women [52] [mean body mass index (BMI) (SD) 34.3 (3.6) kg/m^2^]). The median day that dose adjustment was permitted was on Day 6 after the start of treatment. 13 studies were conducted in Europe, two studies were international (Europe, USA, Canada, Brazil and Russia), one study was conducted in Brazil, one in the USA and one in Australia. Baseline patient characteristics, including age, BMI, AMH, AFC and serum FSH are summarized in Supplementary Table 2.

In the 18 studies, GONAL-f® (follitropin alfa, r-hFSH; Merck KGaA, Darmstadt, Germany) was the most frequently used FSH, followed by Puregon® (follitropin-b, r-hFSH, Merck Sharp & Dohme B. V, The Netherlands), being used in 13 and 6 studies, respectively, with some studies using both treatments. Two studies used biosimilars: one study used Bemfola® (follitropin alfa, Gedeon Richter Plc, Hungary) and one study used Ovaleap® (follitropin alfa, Theramex Ireland Limited, Ireland). The types of FSH used for treatment and any pre-treatment biomarkers measured are summarized in Supplementary Table 3.

### Incidence of dose adjustment

**Fig. 3:**
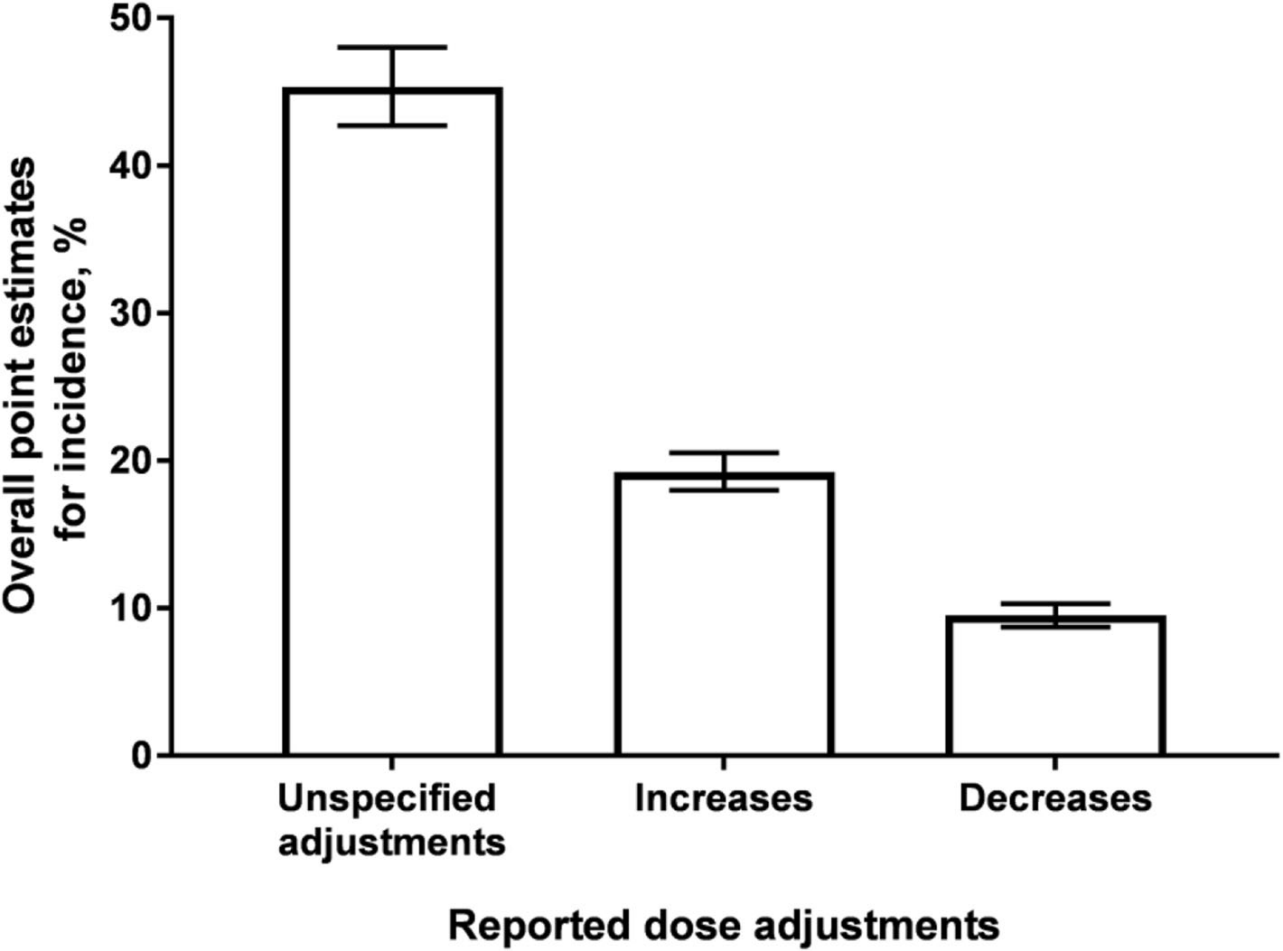
Collective point estimates for incidence of dose adjustment based on data from pooled cycles

Overall, the pooled point estimates for incidence (95% CI) for unspecified dose adjustment, dose increases, and dose decreases were 45.3% (42.7, 48.0), 19.2% (18.0, 20.5), and 9.5% (8.7, 10.3), respectively (Fig. 3).

**Fig. 4:**
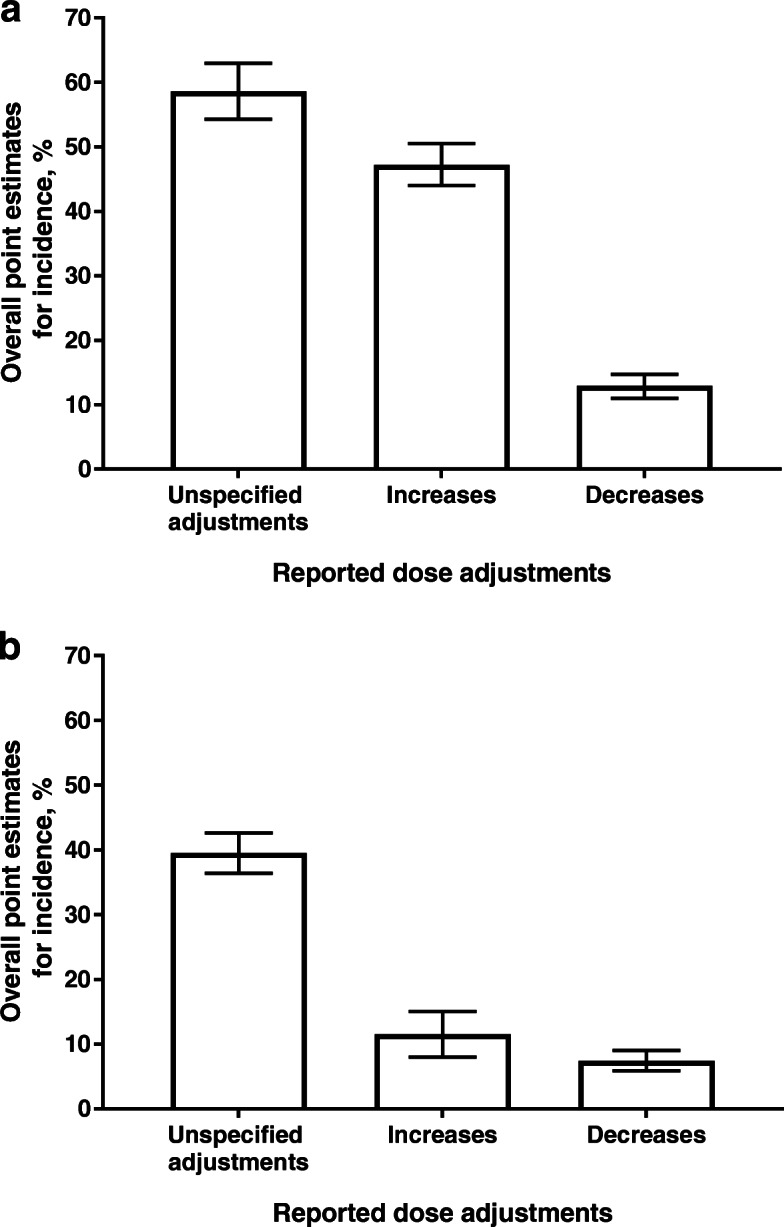
Point estimates for incidence of dose adjustment according GnRH protocol: **a** agonist or **b** antagonist*.* Three studies did not specify the GnRH protocol used or used a mixture of agonists and antagonists, and these data were excluded from this subanalysis

When data were analyzed according to the GnRH protocol used, dose adjustment was more frequent with GnRH agonists than GnRH antagonists. The respective point estimates for incidence (95% CI) for unspecified dose adjustment, dose increases, and dose decreases were 58.6% (54.3, 63.0), 47.2% (44.0, 50.5), and 12.9% (11.0, 14.7) for GnRH agonists, and 39.5% (36.4, 42.6), 11.5% (8.0, 15.0), and 7.4% (5.9, 9.0) for GnRH antagonists (Fig. 4**)**.

When looking at individual studies rather than pooled data, the lowest and highest point estimates for incidence (95% CI) in an individual study were 26.8% (13.3, 40.4) and 72.7% (64.0, 81.5), respectively, for an unspecified dose adjustment; 3.0% (0.8, 5.3) and 58.5% (45.2, 71.8), respectively, for dose increases; and 1.9% (−1.8, 5.5) and 53.4% (47.0, 59.8), respectively, for dose decreases. Point estimates for individual studies are shown in Table 1.

**Table 1:** Point estimates for incidence of unspecified dose adjustment, dose increase and dose decrease for individual studies included in the systematic review

Study	Study arm^**a**^	Unspecified dose adjustment (direction not specified), point estimate (%) for incidence (95% CI)	Dose increase, point estimate (%) for incidence (95% CI)	Dose decrease, point estimate (%) for incidence (95% CI)
**Allegra 2017** [48]	**Control (*N*** **= 99)**	72.7 (64.0, 81.5)	–	–
	**Nomogram**^b^ **(*N*** **= 92)**	60.9 (50.9, 70.8)	–	–
**Buhler 2014** [49]	**(*N*** **= 2074)**	–	4.8 (3.9, 5.7)	3.7 (2.9, 4.5)
**Magnusson 2017** [58]	**AMH (*N*** **= 152)**	54.6 (46.7, 62.5)	–	–
	**Non-AMH (*N*** **= 155)**	52.3 (44.4, 60.1)	–	–
**Espinós 2017**^**c**^ [52]	**(*N*** **= 41)**	26.8 (13.3, 40.4)	–	–
**Nyboe Andersen 2017** [9]	**r-hFSH (*N*** **= 661)**	36.8 (33.1, 40.4)	–	–
**Rettenbacher 2015** [62]	**Bemfola (*N*** **= 220)**	–	–	17.3 (12.3, 22.3)
	**GONAL-f (*N*** **= 113)**	–	–	14.2 (7.7, 20.6)
**Strowitzki 2016** [63]	**Ovaleap (*N*** **= 153)**	–	35.9 (28.3, 43.6)	15.0 (9.4, 20.7)
	**GONAL-f (*N*** **= 146)**	–	43.2 (35.1, 51.2)	15.1 (9.3, 20.9)
**Devroey 2012** [50]	**r-hFSH (*N*** **= 375)**		24.8 (20.4, 29.2)	2.1 (0.7, 3.6)
**Durnerin 2008** [51]	**Control (*N*** **= 49)**	–	55.1 (41.2, 69.0)	2.0 (−1.9, 6.0)
	**r-hLH pretreat (*N*** **= 53)**	–	54.7 (41.3, 68.1)	3.8 (−1.4, 8.9)
**Esteves 2009** [53]	**r-hFSH (*N*** **= 236)**	–	–	53.4 (47.0, 59.8)
**Devroey 2009** [54]	**r-hFSH (*N*** **= 750)**	–	–	8.4 (6.4, 10.4%)
**Freiesleben 2008** [55]	**r-hFSH (*N*** **= 159)**	44.0 (36.3, 51.7)	–	–
**Kyrou 2009** [56]	**r-hFSH (*N*** **= 230)**	–	3.0 (0.8, 5.3)	7.0 (3.7, 10.2)
**Lossl 2008** [57]	**Androgen priming (N = 53)**	–	58.5 (45.2, 71.8)	1.9 (−1.8, 5.5)
	**Control (*N*** **= 50)**	–	52.0 (38.2, 65.8)	2.0 (−1.9, 5.9)
**Nakhuda 2010**^**d**^ [59]	**(*N*** **= 104)**	–	10.6 (4.7, 16.5)	35.6 (26.4, 44.8)
**Nyboe Andersen 2008** [60]	**r-hFSH (*N*** **= 261)**	–	51.7 (45.7, 57.8)	11.5 (7.6, 15.4)
	**r-hFSH + r-hLH (*N*** **= 265)**	–	48.7 (42.7, 54.7)	11.3 (7.5, 15.1)
**Requena 2010** [61]	**r-hFSH + HP-hMG (*N*** **= 46)**	–	32.6 (19.1, 46.2)	–
	**r-hFSH (N = 46)**	–	32.6 (19.1, 46.2)	–
**Yovich 2012** [40]	**FSH dose < 100 IU (*N*** **= 47)**	–	53.2 (38.9, 67.5)	–

*AMH* anti-Müllerian hormone, *FSH* follicle stimulating hormone, *r-hFSH* recombinant-human follicle stimulating hormone, *r-hLH* recombinant-human luteinizing hormone, *HP-hMG* highly purified human menopausal gonadotropin

^a^Only study arms using recombinant FSH were included in the data analysis; ^b^FSH starting dose set using a nomogram based on age, serum Day 3 FSH and AMH; ^c^Study in obese women; ^d^Study in oocyte donors

## Discussion

In clinical trials in women receiving ART treatment that allowed dose adjustment within the study protocol, gonadotropin dose adjustment during treatment was reported in 45% of cycles evaluated (direction unspecified), with dose increases reported more frequently than dose decreases (19% vs 10%, respectively). These results highlight that dose adjustment, when allowed according to study protocol, are used in published clinical trials of patients receiving ART treatment. It can be postulated that these dose adjustments occurred where healthcare professionals thought modification of the starting dose during treatment would result in improved outcomes. For example, in patients with unexpected poor ovarian response, increasing the gonadotropin dose may improve reproductive outcomes (by optimizing the number and quality of oocytes, resulting in good quality embryos) and reduce risks (such as reduced cycle cancellation rates), whereas in patients with unexpected hyper ovarian response, decreasing the gonadotropin dose may reduce the risk of OHSS.

As only two studies in our systematic review reported dose adjustment in patients with predicted poor response (compared with three studies in patients with normal response, six studies in patients with hyper-response and seven studies of unknown response levels [AFC/AMH data not reported]), it could be postulated that the higher frequency of dose increases compared with dose decreases observed in our study were in patients with unexpected poor or insufficient response to ovarian stimulation. However, it is unclear whether dose adjustment during stimulation cycles per se or the final total dose of gonadotropin resulting from dose adjustment during ovarian stimulation affects the clinical and OHSS outcomes. The studies included in our systematic review were not specifically selected for the clinical outcomes; consequently, only three reported direct comparisons of outcomes in the constant dose versus the dose adjustment groups [9, 40, 59]. Of these studies, total dose was only reported in one study [9], but the results of this study have been challenged [3, 64, 65] as both treatment arms not only differed with respect to dose adjustment during ovarian stimulation, but also were different in the type of FSH preparation used, and in calculation and choice of FSH starting dose. Of the studies on which the ESHRE Guidelines on dose adjustment were based, only two reported the total dose [66, 67], and the total dose reflected the direction of adjustment (i.e., lower total dose when the dose was reduced [66] and higher total dose when the dose was increased during ovarian stimulation [67], as can be expected). Therefore, additional studies are required to assess whether total dose or dose adjustment or both are important in affecting safety (OHSS risk) and efficacy (cycle cancellation risk and reproductive outcomes per started cycle).

Overall, of the 18 studies included in the systematic review, different GnRH analogs were used: eight used a long agonist, eight used an antagonist, one used a mixture and one was not specified. When the three studies in which direct comparisons of outcomes in the dose adjustment and constant dose groups were reported, one used an agonist (dose increases and decreases) [59], one used an antagonist (dose adjustment unspecified) [9] and one used agonist/antagonist (dose increases) [68]. A subanalysis examining dose adjustment according to the GnRH protocol used revealed that unspecified dose adjustment was more common in patients receiving a GnRH agonist than antagonist, being reported in approximately 59% versus 40% of cycles, respectively. Dose increases were also more frequent with GnRH agonists than antagonists (47% vs 12%, respectively), as were dose decreases (13% vs 7%, respectively). GnRH antagonists competitively bind to the GnRH receptor, causing immediate and rapid gonadotrophin suppression, and their onset of action is significantly faster than that of GnRH agonists, which in contrast suppress gonadotrophin release by desensitizing the pituitary receptors [69, 70]. In addition, GnRH antagonist suppression of gonadotropin secretion is rapidly reversible [71]. As a result, GnRH antagonist treatment is associated with a shorter duration of ovarian stimulation than with a GnRH agonist, as well as lower OHSS rates [72–75]. In a Cochrane review of 73 RCTs, Al-Inany et al. reported that antagonists were associated with a lower incidence of OHSS (odds ratio [OR] 0.61 [95% CI 0.51, 0.72]) and cycle cancellation due to a high OHSS risk (OR 0.47 [95% CI 0.32, 0.69]) compared with agonists [76]. Physicians may therefore be more likely to adjust the FSH dose in those patients receiving a GnRH agonist compared with a GnRH antagonist, in order to reduce the OHSS risk, which is supported by the findings of this systematic review. Of the included studies, 16 used hCG triggering, one used an agonist trigger (antagonist down-regulation from Day 6) [9] and one used hCG/agonist trigger (in antagonist cycles with > 20 follicles; long down regulation flare cycle agonist/antagonist conversion with estrogen priming or antagonist, according to local practice) [68]) (data not shown). The GnRH agonist protocol, which is also associated with an increased risk of OHSS when compared to a GnRH antagonist protocol [77], cannot be used in conjunction with a GnRH agonist trigger. Furthermore, dose increases were reported more frequently than dose decreases in patients receiving GnRH agonists (47% vs 13%) and GnRH antagonists (12% vs 7%), in-line with the overall population, suggesting that more patients may have had unexpected poor or insufficient responses, resulting in physicians acting to increase the FSH dose in these patients.

Dose adjustment during treatment has been observed in an analysis of a routine care setting from a large US insurance database [29]. This analysis reported the occurrence of dose adjustment of r-hFSH and the characteristics of patients receiving r-hFSH for COS as part of IVF between July 2009 and December 2016. In this analysis, 41% of 13,823 cycles from 23,582 patients undergoing IVF included ≥ 1 dose adjustments, with a greater proportion receiving dose decreases than dose increases (63 vs 57% of cycles). Patients who received dose adjustment were older, had a higher AFC and AMH, and a lower Day 3 FSH compared with those who did not receive a dose adjustment. Patients who received a dose adjustment were also more likely to have a diagnosis of diminished ovarian reserve and PCOS than those who received a fixed dose.

A lower proportion of patients received a dose adjustment in our review of clinical trials compared with the analysis of routine clinical practice in the USA [29] (with unspecified dose adjustments, increases and decreases reported in 45, 19, and 10% of cycles in our study vs 41, 57, and 63%, respectively, in the US study). This is not surprising as more than half of the studies (10/18) our review identified were RCTs, which have highly selected patient populations based on their inclusion and exclusion criteria (for example, none of the RCTs identified in our review comprised patients with predicted poor response based on their AFC/AMH levels, whereas 10 to 20% of patients undergoing IVF are reported to have poor ovarian responses [78]); accordingly it can be anticipated that dose adjustment is more controlled and less freely available to physicians participating in RCTs compared with the routine care setting in the USA. Furthermore, the majority of studies included in our systematic review were from Europe (> 70%), likely resulting in differences in clinical practices between our study and the US analysis [29].

Although the ovarian response markers used to evaluate the need for dose adjustment during treatment were not assessed in this analysis, dose adjustment during ovarian stimulation is usually based on ovarian response measured by ultrasound and/or hormonal monitoring. Estradiol levels and follicular size, as measured by ultrasound, are directly linked to progesterone production, with progesterone rise associated with a negative effect on pregnancy rates [79]. Adapting the stimulation dose during the late follicular phase according to the patient’s response is sufficient to prevent this progesterone increase, as demonstrated by Lawrenz et al. [80] in a combined post-hoc analysis of the ENGAGE and PURSUE trials. In a subgroup analysis comparing the occurrence of premature progesterone rise in the women treated corifollitropin alfa with or without daily rFSH after Day 8, there was a significant increase in premature progestin rise in those with additional rFSH compared with those without (20.8% vs 5.4%; *p* < 0.001), confirming that enhanced FSH stimulation towards the end of the follicular phase was responsible for the premature progestin rise [80]. However, the ESHRE guidelines suggest that the addition of estradiol to ultrasound testing during ovarian stimulation does not appear to increase the probability of pregnancy or the number of oocytes retrieved, or to decrease the probability of OHSS compared with ultrasound alone [14]. It is important to note, however, that this recommendation was labeled by ESHRE as ‘conditional’ (‘*probably recommend*’), for which ESHRE suggests that clinicians should recognize that different choices will be appropriate for individual patients, as opposed to recommendations labeled as ‘strong’ (‘*recommend*’), for which ESHRE suggests most individuals should receive the intervention. Furthermore, this guidance was based on the Cochrane review by Kwan et al. [81], and there is a major concern over the validity of this recommendation for OHSS risk management: only 781 women from 6 studies were included for analysis with ~ 4% OHSS rate, a point that Kwan et al. acknowledge, along with the need for a confirmatory large well-designed RCT. The addition of estradiol to ultrasound monitoring may be beneficial as precautionary good clinical practice and as a confirmatory test in women at high risk of OHSS [81], and it is common practice in many countries/regions to have routine monitoring with frequent ultrasound and/or estradiol measurements in women at high risk of OHSS [26, 82]. Furthermore, despite the conditional recommendation by ESHRE that an inclusion of an extended hormonal panel (estradiol, progesterone and luteinizing hormone [LH]) measures to ultrasound testing during ovarian stimulation does not appear to increase the probability of pregnancy or to decrease the probability of OHSS or cycle cancellation for high responders [14], this was based on studies with a small sample size which reported only a few cases of OHSS [83, 84]. Accordingly, a proviso that “the decision on timing of triggering in relation to follicle size is multi-factorial, taking into account the size of the growing follicle cohort, the hormonal data on the day of pursued trigger … …” was stipulated in the guidelines [14].

Few clinical trials have compared the effects of FSH dose adjustment during a treatment cycle with a fixed starting FSH dose; therefore, the actual effect of these dose adjustments on outcomes have not been fully investigated and cannot be determined from the data obtained by this systematic review. In a small RCT in patients with expected poor response, Klinkert et al. (2005) compared standard 150 IU rFSH dose (*n* = 26; 150 IU with the possibility of the dose being increased to 300 IU during treatment) with 300 IU rFSH dose (n = 26; 300 IU fixed dose). Nine patients (35%) in the standard rFSH (150 IU) group received dose adjustments due to an insufficient response, resulting in the dose being increased to 300 IU, but no improvement in ovarian responses were reported in these patients [85]. However, this was a small study and was not designed to assess dose adjustments during treatment, but rather to evaluate standard starting dose (150 IU) versus higher (300 IU) starting doses; therefore, it is impossible to draw conclusions concerning dose adjustments from this study. Previous studies assessing dose adjustment of human menopausal gonadotropin (hMG, containing FSH and LH activity) in patients with poor ovarian response [66] and in unselected patients [23, 86] have shown non-inferiority in terms of pregnancy rates compared with fixed hMG dose, suggesting limited benefit in adjusting the hMG dose during treatment. Based on this evidence, the ESHRE guidelines for ovarian stimulation for IVF/ ICSI states that adjustment (increase or decrease) of the gonadotropin dose in the mid-stimulation phase during ovarian stimulation is probably not recommended [14]. However, the recommendations based on such studies can be confounding, as evidence only indicates that there is no difference in assessed efficacy outcomes and does not consider additional relevant outcomes and risks that are relevant to patients (cycle cancellation, OHSS), costs or patient preferences. Indeed, in the studies on which these guidelines were based, no differences were reported for clinical outcomes (e.g., embryo number [23, 66, 67, 86], clinical pregnancy rates [23, 86, 87] or ongoing pregnancy rates [23]; live birth rates were not reported in any of the studies), although in a retrospective chart review of a mixed population [23], the number of oocytes retrieved was 9.7 (0.3) in the patients with a constant dose versus 13.4 (0.7) in the patients with dose decrease (*p* < 0.001). Furthermore, several of these studies also reported non-significant trends towards favorable outcomes in patients with dose adjustments compared with those with constant doses (e.g., higher implantation rates with dose increase [86] and with dose increase and decrease [23], higher clinical pregnancy rates with dose increase [86], lower cycle cancellation with dose decease before coasting [87] and lower incidence of moderate OHSS with dose decrease before coasting [87]). In the two studies in our systematic review for which meaningful comparisons could be made, there was a significant difference in the mean (SD) number of oocytes retrieved (19.38 [1.18] for no dose change, 16.75 [2.5] with a dose increase and 26.89 [1.64] after dose decrease; *p* < 0.001) [59] and a trend towards higher oocyte numbers (8.5 [4.5] in constant dose group and 9.3 [6.3] in the dose increase group [40]), respectively. Only one case of severe OHSS was reported with dose increase, which was attributed to a violation of the PIVET algorithm [40]. Finally, the findings of the recent OPTIMIST trial could also be considered, as dose adjustment between cycles was permitted in both treatment arms to a maximum of 50 IU/ day, which could be considered as a proxy for dose individualization, as adjustments were not allowed to be made for any patients included in the study arms [21, 27]. Although no benefit of dose individualization based on AFC was reported for cumulative live birth outcomes in the poor responders, the reported data showed benefits of dose individualization on outcomes that are directly modifiable by dose adjustment (e.g., cycle cancellation, poor response, oocyte yield, number of fresh embryos and fresh transfer rate) [88, 89]. Therefore, larger properly designed studies are still needed to evaluate the differences in efficacy outcomes with dose adjustment during treatment compared with fixed gonadotropin dosing during ovarian stimulation. Coasting (withholding gonadotropin dose administration for ≥ 1 day) can also be considered as a form of dose reduction during a stimulation cycle. In a systematic review of 493 patients in 12 studies, Delvigne and Rozenberg concluded that although the data were highly heterogeneous with respect to patient numbers and characteristics, as well as stimulation and coasting procedures, coasting decreased the risk of OHSS in high risk patients, although did not avoid the risk altogether [90]. A later Cochrane Review of 8 RCTs reported only low-level evidence based on data from 702 women at high risk of developing OHSS that coasting reduced moderate or severe OHSS more than no coasting [91]; however, only two of the included studies directly compared coasting with no coasting for the occurrence of OHSS [92, 93], both reporting lower rates of OHSS in the coasting groups than in the non-coasting groups. Despite this evidence, the American Society for Reproductive Medicine (ASRM) guidelines suggest there is insufficient evidence to recommend coasting for the prevention of OHSS. Furthermore, the effects of coasting on pregnancy outcomes are unclear [82].

Limitations of this systematic review included the fact that we specifically looked at r-hFSH in order to limit the scope of the search, which, even after restricting to r-hFSH, identified over 1400 publications for initial screening. Furthermore, we only included articles written in English, used data from a restricted time period, and only used PubMed for database searches. As this was a systematic review of published data, the effect of dose adjustment on outcomes could not be evaluated.

## Conclusions

According to this systematic review, in studies in which r-hFSH dose adjustment was allowed and reported, the estimated incidence of r-hFSH dose adjustment during ovarian stimulation was up to 45%, with dose increases occurring more commonly than dose decreases. Dose adjustment was more frequent in patients receiving a GnRH agonist than a GnRH antagonist, and was reported in patients with a predicted poor, normal, or high response. In patients with poor ovarian response, increasing the FSH dose during ovarian stimulation may increase the number of oocytes retrieved and reduce the risk of cycle cancellation due to insufficient response; conversely, in patients predicted to have a hyper-response to FSH, decreasing the FSH dose may reduce the risk of OHSS and related risk for cycle cancellation. It may be hypothesized that healthcare providers consider dose adjustment during ovarian stimulation worthwhile for improving treatment outcomes and/or reducing risks, and this is reflected in clinical trial designs that allow dose adjustment. The incidence of this dose adjustment in routine clinical practice and its impact on clinical outcomes requires further evaluation.

## Supplementary Information

**Additional file 1.**

## Data Availability

All data analysed during this study are included in this published article and its supplementary information files.

## Abbreviations

FSH: follicle-stimulating hormone
COS: controlled ovarian stimulation
ART: assisted reproductive technology
r-hFSH: recombinant-human FSH
CI: confidence interval
IVF: in vitro fertilization
GnRH: gonadotropin-releasing hormone
ICSI: intracytoplasmic sperm injection
ESHRE: European Society of Human Reproduction and Embryology
POR: poor ovarian response
OHSS: over hyperstimulation syndrome
AMH: anti-Müllerian hormone
AFC: antral follicle count
OI: ovulation induction
PCOS: polycystic ovary syndrome
SD: standard deviation
BMI: body mass index
ASRM: American Society of Reproductive Medicine

## References

[1] Ruiz-Alonso M, Galindo N, Pellicer A, Simon C (2014). What a difference two days make: "personalized" embryo transfer (pET) paradigm: a case report and pilot study. Hum Reprod.

[2] Lawrenz B, Samir S, Garrido N, Melado L, Engelmann N, Fatemi HM (2018). Luteal Coasting and Individualization of Human Chorionic Gonadotropin Dose after Gonadotropin-Releasing Hormone Agonist Triggering for Final Oocyte Maturation-A Retrospective Proof-of-Concept Study. Front Endocrinol (Lausanne).

[3] Mol BW, Bossuyt PM, Sunkara SK, Garcia Velasco JA, Venetis C, Sakkas D, Lundin K, Simón C, Taylor HS, Wan R, Longobardi S, Cottell E, D'Hooghe T (2018). Personalized ovarian stimulation for assisted reproductive technology: study design considerations to move from hype to added value for patients. Fertil Steril.

[4] Lunenfeld B, Bilger W, Longobardi S, Alam V, D'Hooghe T, Sunkara SK (2019). The Development of Gonadotropins for Clinical Use in the Treatment of Infertility. Front Endocrinol (Lausanne).

[5] EMA. Puregon: Sumamry of product characteristics. 2021.

[6] EMA. Gonal-f: summary of product characteristics. 2020.

[7] Alviggi C, Humaidan P, Ezcurra D (2012). Hormonal, functional and genetic biomarkers in controlled ovarian stimulation: tools for matching patients and protocols. Reprod Biol Endocrinol.

[8] Lensen SF, Wilkinson J, Leijdekkers JA, La Marca A, Mol BWJ, Marjoribanks J, et al. Individualised gonadotropin dose selection using markers of ovarian reserve for women undergoing in vitro fertilisation plus intracytoplasmic sperm injection (IVF/ICSI). Cochrane Database Syst Rev. 2018;2(2):Cd012693. 10.1002/14651858.CD012693.pub2 PMC6491064 29388198

[9] Nyboe Andersen A, Nelson SM, Fauser BC, Garcia-Velasco JA, Klein BM, Arce JC (2017). Individualized versus conventional ovarian stimulation for in vitro fertilization: a multicenter, randomized, controlled, assessor-blinded, phase 3 noninferiority trial. Fertility Sterility.

[10] Nelson SM (2013). Biomarkers of ovarian response: current and future applications. Fertil Steril.

[11] Sighinolfi G, Grisendi V, La Marca A (2017). How to personalize ovarian stimulation in clinical practice. J Turk Ger Gynecol Assoc.

[12] Alviggi C, Conforti A, Esteves SC, Vallone R, Venturella R, Staiano S, et al. Understanding Ovarian Hypo-Response to Exogenous Gonadotropin in Ovarian Stimulation and Its New Proposed Marker-The Follicle-To-Oocyte (FOI) Index. Front Endocrinol (Lausanne). 2018;9:589. 10.3389/fendo.2018.00589 PMC6199413 30386293

[13] Lunenfeld B, Bilger W, Longobardi S, Kirsten J, D'Hooghe T, Sunkara SK (2019). Decision points for individualized hormonal stimulation with recombinant gonadotropins for treatment of women with infertility. Gynecol Endocrinol.

[14] Bosch E, Broer S, Griesinger G, Grynberg M, Humaidan P, Kolibianakis E (2020). ESHRE guideline: ovarian stimulation for IVF/ICSI. Hum Reprod Open.

[15] La Marca A, Sighinolfi G, Radi D, Argento C, Baraldi E, Artenisio AC (2010). Anti-Mullerian hormone (AMH) as a predictive marker in assisted reproductive technology (ART). Hum Reprod Update.

[16] Nelson SM, Yates RW, Fleming R (2007). Serum anti-Mullerian hormone and FSH: prediction of live birth and extremes of response in stimulated cycles--implications for individualization of therapy. Hum Reprod.

[17] Sadeghi MR (2017). How can personalized medicine improve assisted reproduction technology outcomes?. J Reprod Infertil.

[18] La Marca A, Papaleo E, Grisendi V, Argento C, Giulini S, Volpe A (2012). Development of a nomogram based on markers of ovarian reserve for the individualisation of the follicle-stimulating hormone starting dose in in vitro fertilisation cycles. Bjog..

[19] Rose TH, Roshammar D, Erichsen L, Grundemar L, Ottesen JT (2016). Characterisation of population pharmacokinetics and endogenous follicle-stimulating hormone (FSH) levels after multiple dosing of a recombinant human FSH (FE 999049) in healthy women. Drugs R D.

[20] Humaidan P, Alviggi C, Fischer R, Esteves SC (2016). The novel POSEIDON stratification of 'Low prognosis patients in Assisted Reproductive Technology' and its proposed marker of successful outcome. F1000Res.

[21] van Tilborg TC, Oudshoorn SC, Eijkemans MJC, Mochtar MH, van Golde RJT, Hoek A (2017). Individualized FSH dosing based on ovarian reserve testing in women starting IVF/ICSI: a multicentre trial and cost-effectiveness analysis. Hum Reprod.

[22] Kovacs P, Sajgo A, Kaali SG, Pal L (2012). Detrimental effects of high-dose gonadotropin on outcome of IVF: making a case for gentle ovarian stimulation strategies. Reprod Sci.

[23] Martin JR, Mahutte NG, Arici A, Sakkas D (2006). Impact of duration and dose of gonadotrophins on IVF outcomes. Reprod Biomed Online.

[24] Lekamge DN, Lane M, Gilchrist RB, Tremellen KP (2008). Increased gonadotrophin stimulation does not improve IVF outcomes in patients with predicted poor ovarian reserve. J Assist Reprod Genet.

[25] Pal L, Jindal S, Witt BR, Santoro N (2008). Less is more: increased gonadotropin use for ovarian stimulation adversely influences clinical pregnancy and live birth after in vitro fertilization. Fertil Steril.

[26] Humaidan P, Nelson SM, Devroey P, Coddington CC, Schwartz LB, Gordon K (2016). Ovarian hyperstimulation syndrome: review and new classification criteria for reporting in clinical trials. Hum Reprod.

[27] Oudshoorn SC, van Tilborg TC, Eijkemans MJC, Oosterhuis GJE, Friederich J, van Hooff MHA, et al. Individualized versus standard FSH dosing in women starting IVF/ICSI: an RCT. Part 2: The predicted hyper responder. Hum Reprod. 2017;32(12):2506–14. 10.1093/humrep/dex319 29121269

[28] Broekmans FJ. Individualization of FSH Doses in Assisted Reproduction: Facts and Fiction. Front Endocrinol (Lausanne). 2019;10:181. 10.3389/fendo.2019.00181 PMC6497745 31080437

[29] Mahony MH, B. Richter, K. D'Hooghe, T. Abstracts of the 34th Annual Meeting of the European Society of Human Reproduction and Embryology (P^−659^); Occurrence and characteristics of recombinant human follicle-stimulating hormone (r-hFSH) dose adjustments during ovarian stimulation in a real-world US database study of 33,962 IVF patient cycles. Hum Reprod. 2018;33(suppl_1):i444.

[30] FDA. Highlights of prescribing information for Gonal-f RFF Redi-ject (follitropin alfa injection) 2013 [Available from: https://www.accessdata.fda.gov/drugsatfda_docs/label/2013/021684s036lbl.pdf.

[31] FDA. Highlights of prescribing information for Follistrim AQ Cartridge (follitropin beta injection) 2004 [Available from: https://www.accessdata.fda.gov/drugsatfda_docs/label/2011/021211s011lbl.pdf.

[32] Ledger WL, Fauser BC, Devroey P, Zandvliet AS, Mannaerts BM (2011). Corifollitropin alfa doses based on body weight: clinical overview of drug exposure and ovarian response. Reprod Biomed Online.

[33] Abbotts C, Salgado-Braga C, Audibert-Gros C (2011). A redesigned follitropin alfa pen injector for infertility: results of a market research study. Patient Prefer Adherence.

[34] Jeannerot F, Cusin A, Schertz J (2016). Dose accuracy of the redesigned follitropin alfa pen injector for infertility treatment. Expert Opin Drug Deliv.

[35] Jeannerot F, Stüdeli T, Gunther-LaVergne L, Hirning D, Schertz J (2016). Usability engineering study in the European Union of a redesigned follitropin alfa pen injector for infertility treatment. Expert Opin Drug Deliv.

[36] Longobardi S, Seidler A, Martins J, Beckers F, MacGillivray W, D'Hooghe T (2019). An evaluation of the use and handling errors of currently available recombinant human follicle-stimulating hormone pen injectors by women with infertility and fertility nurses. Expert Opin Drug Deliv.

[37] Schertz J, Worton H (2017). Patient evaluation of the redesigned follitropin alfa pen injector. Expert Opin Drug Deliv.

[38] Schertz J, Worton H (2018). Nurse evaluation of the redesigned fertility pen injector: a questionnaire-based observational survey. Expert Opin Drug Deliv.

[39] Olivennes F, Trew G, Borini A, Broekmans F, Arriagada P, Warne DW, Howles CM (2015). Randomized, controlled, open-label, non-inferiority study of the CONSORT algorithm for individualized dosing of follitropin alfa. Reprod Biomed Online.

[40] Yovich J, Stanger J, Hinchliffe P (2012). Targeted gonadotrophin stimulation using the PIVET algorithm markedly reduces the risk of OHSS. Reprod Biomed Online.

[41] Yovich JL, Alsbjerg B, Conceicao JL, Hinchliffe PM, Keane KN (2016). PIVET rFSH dosing algorithms for individualized controlled ovarian stimulation enables optimized pregnancy productivity rates and avoidance of ovarian hyperstimulation syndrome. Drug Des Devel Ther.

[42] Drakopoulos P, Blockeel C, Stoop D, Camus M, de Vos M, Tournaye H, et al. Conventional ovarian stimulation and single embryo transfer for IVF/ICSI. How many oocytes do we need to maximize cumulative live birth rates after utilization of all fresh and frozen embryos? Hum Reprod. 2016;31(2):370–6. 10.1093/humrep/dev316 26724797

[43] Malchau SS, Henningsen AA, Forman J, Loft A, Nyboe Andersen A, Pinborg A. Cumulative live birth rate prognosis based on the number of aspirated oocytes in previous ART cycles. Hum Reprod. 2019;34(1):171–80. 10.1093/humrep/dey341 30541039

[44] Polyzos NP, Sunkara SK. Sub-optimal responders following controlled ovarian stimulation: an overlooked group? Hum Reprod. 2015;30(9):2005–8. 10.1093/humrep/dev149 26202582

[45] Delvigne A, Rozenberg S (2002). Epidemiology and prevention of ovarian hyperstimulation syndrome (OHSS): a review. Hum Reprod Update.

[46] Gera PS, Tatpati LL, Allemand MC, Wentworth MA, Coddington CC (2010). Ovarian hyperstimulation syndrome: steps to maximize success and minimize effect for assisted reproductive outcome. Fertil Steril.

[47] Grant MJ, Booth A (2009). A typology of reviews: an analysis of 14 review types and associated methodologies. Health Info Libr J.

[48] Allegra A, Marino A, Volpes A, Coffaro F, Scaglione P, Gullo S, la Marca A (2017). A randomized controlled trial investigating the use of a predictive nomogram for the selection of the FSH starting dose in IVF/ICSI cycles. Reprod Biomed Online.

[49] Buhler K, Naether OG, Bilger W (2014). A large, multicentre, observational, post-marketing surveillance study of the 2:1 formulation of follitropin alfa and lutropin alfa in routine clinical practice for assisted reproductive technology. Reprod Biol Endocrinol.

[50] Devroey P, Pellicer A, Nyboe Andersen A, Arce JC (2012). Menopur in Gn RHACwSETTG. A randomized assessor-blind trial comparing highly purified hMG and recombinant FSH in a GnRH antagonist cycle with compulsory single-blastocyst transfer. Fertil Steril.

[51] Durnerin CI, Erb K, Fleming R, Hillier H, Hillier SG, Howles CM, et al. Effects of recombinant LH treatment on folliculogenesis and responsiveness to FSH stimulation. Hum Reprod. 2008;23(2):421–6. 10.1093/humrep/dem388 18084048

[52] Espinós JJ, Polo A, Sanchez-Hernandez J, Bordas R, Pares P, Martinez O (2017). Weight decrease improves live birth rates in obese women undergoing IVF: a pilot study. Reprod Biomed Online.

[53] Esteves SC, Schertz JC, Verza S, Schneider DT, Zabaglia SF (2009). A comparison of menotropin, highly-purified menotropin and follitropin alfa in cycles of intracytoplasmic sperm injection. Reprod Biol Endocrinol.

[54] Devroey P, Boostanfar R, Koper NP, Mannaerts BM, Ijzerman-Boon PC, Fauser BC. A double-blind, non-inferiority RCT comparing corifollitropin alfa and recombinant FSH during the first seven days of ovarian stimulation using a GnRH antagonist protocol. Hum Reprod. 2009;24(12):3063–72. 10.1093/humrep/dep291 PMC2777786 19684043

[55] Freiesleben NL, Lossl K, Bogstad J, Bredkjaer HE, Toft B, Loft A (2008). Predictors of ovarian response in intrauterine insemination patients and development of a dosage nomogram. Reprod Biomed Online.

[56] Kyrou D, Popovic-Todorovic B, Fatemi HM, Bourgain C, Haentjens P, Van Landuyt L, et al. Does the estradiol level on the day of human chorionic gonadotrophin administration have an impact on pregnancy rates in patients treated with rec-FSH/GnRH antagonist? Hum Reprod. 2009;24(11):2902–9. 10.1093/humrep/dep290 19671625

[57] Lossl K, Andersen CY, Loft A, Freiesleben NL, Bangsboll S, Andersen AN. Short-term androgen priming by use of aromatase inhibitor and hCG before controlled ovarian stimulation for IVF. A randomized controlled trial. Hum Reprod. 2008;23(8):1820–9. 10.1093/humrep/den131 18487212

[58] Magnusson A, Nilsson L, Olerod G, Thurin-Kjellberg A, Bergh C. The addition of anti-Mullerian hormone in an algorithm for individualized hormone dosage did not improve the prediction of ovarian response-a randomized, controlled trial. Hum Reprod. 2017;32(4):811–9. 10.1093/humrep/dex012 28175316

[59] Nakhuda GS, Douglas NC, Thornton MH, Guarnaccia MM, Lobo R, Sauer MV (2010). Anti-Mullerian hormone testing is useful for individualization of stimulation protocols in oocyte donors. Reprod Biomed Online.

[60] Nyboe Andersen A, Humaidan P, Fried G, Hausken J, Antila L, Bangsboll S (2008). Recombinant LH supplementation to recombinant FSH during the final days of controlled ovarian stimulation for in vitro fertilization. A multicentre, prospective, randomized, controlled trial. Hum Reprod.

[61] Requena A, Landeras JL, Martinez-Navarro L, Calatayud C, Sanchez F, Maldonado V (2010). Could the addition of hp-hMG and GnRH antagonists modulate the response in IVF-ICSI cycles?. Hum Fertil (Camb).

[62] Rettenbacher M, Andersen AN, Garcia-Velasco JA, Sator M, Barri P, Lindenberg S, van der Ven K, Khalaf Y, Bentin-Ley U, Obruca A, Tews G, Schenk M, Strowitzki T, Narvekar N, Sator K, Imthurn B (2015). A multi-Centre phase 3 study comparing efficacy and safety of Bemfola((R)) versus Gonal-f((R)) in women undergoing ovarian stimulation for IVF. Reprod Biomed Online.

[63] Strowitzki T, Kuczynski W, Mueller A, Bias P (2016). Randomized, active-controlled, comparative phase 3 efficacy and safety equivalence trial of Ovaleap(R) (recombinant human follicle-stimulating hormone) in infertile women using assisted reproduction technology (ART). Reprod Biol Endocrinol.

[64] Longobardi S.; D’Hooghe T. Comments on the results of the ESTHER-1 trial (Evidence-based Stimulation Trial with human rFSH in Europe and Rest of world 1). Available at: https://www.fertstertdialog.com/users/16110-fertility-and-sterility/posts/12852-23086. 2017.

[65] Wilkinson J. Comments on the results of the ESTHER-1 trial (Evidence-based Stimulation Trial with human rFSH in Europe and Rest of world 1). Available at: https://www.fertstertdialog.com/users/16110-fertility-and-sterility/posts/12852-23086. 2017.

[66] Cedrin-Durnerin I, Bstandig B, Herve F, Wolf J, Uzan M, Hugues J (2000). A comparative study of high fixed-dose and decremental-dose regimens of gonadotropins in a minidose gonadotropin-releasing hormone agonist flare protocol for poor responders. Fertil Steril.

[67] van Hooff MH, Alberda AT, Huisman GJ, Zeilmaker GH, Leerentveld RA (1993). Doubling the human menopausal gonadotrophin dose in the course of an in-vitro fertilization treatment cycle in low responders: a randomized study. Hum Reprod.

[68] Yovich JL, Hinchliffe PM, Lingam S, Srinivasan S, Keane KN. Adjusting the PIVET rFSH dosing algorithm for the biosimilar Bemfola product. J Fertil In vitro IVF Worldw Reprod Med Genet Stem Cell Biol. 2018;5:3.

[69] Huirne JA, Homburg R, Lambalk CB (2007). Are GnRH antagonists comparable to agonists for use in IVF?. Hum Reprod.

[70] Cheung LP, Lam PM, Lok IH, Chiu TT, Yeung SY, Tjer CC (2005). GnRH antagonist versus long GnRH agonist protocol in poor responders undergoing IVF: a randomized controlled trial. Hum Reprod.

[71] Gordon K, Hodgen GD (1992). GnRH agonists and antagonists in assisted reproduction. Baillieres Clin Obstet Gynaecol.

[72] Al-Inany HG, Youssef MA, Aboulghar M, Broekmans F, Sterrenburg M, Smit J (2011). Gonadotrophin-releasing hormone antagonists for assisted reproductive technology. Cochrane Database Syst Rev.

[73] Lambalk CB, Banga FR, Huirne JA, Toftager M, Pinborg A, Homburg R, van der Veen F, van Wely M (2017). GnRH antagonist versus long agonist protocols in IVF: a systematic review and meta-analysis accounting for patient type. Hum Reprod Update.

[74] Xiao JS, Su CM, Zeng XT (2014). Comparisons of GnRH antagonist versus GnRH agonist protocol in supposed normal ovarian responders undergoing IVF: a systematic review and meta-analysis. PLoS One.

[75] Pundir J, Sunkara SK, El-Toukhy T, Khalaf Y (2012). Meta-analysis of GnRH antagonist protocols: do they reduce the risk of OHSS in PCOS?. Reprod Biomed Online.

[76] Al-Inany HG, Youssef MA, Ayeleke RO, Brown J, Lam WS, Broekmans FJ (2016). Gonadotrophin-releasing hormone antagonists for assisted reproductive technology. Cochrane Database Syst Rev.

[77] Devroey P, Polyzos NP, Blockeel C (2011). An OHSS-Free Clinic by segmentation of IVF treatment. Hum Reprod.

[78] Grisendi V, Mastellari E, La Marca A (2019). Ovarian Reserve Markers to Identify Poor Responders in the Context of Poseidon Classification. Front Endocrinol (Lausanne).

[79] Schneyer AL, Fujiwara T, Fox J, Welt CK, Adams J, Messerlian GM, Taylor AE (2000). Dynamic changes in the intrafollicular inhibin/activin/follistatin axis during human follicular development: relationship to circulating hormone concentrations. J Clin Endocrinol Metab.

[80] Lawrenz B, Labarta E, Fatemi H, Bosch E (2018). Premature progesterone elevation: targets and rescue strategies. Fertil Steril.

[81] Kwan I, Bhattacharya S, Kang A, Woolner A (2014). Monitoring of stimulated cycles in assisted reproduction (IVF and ICSI). Cochrane Database Syst Rev.

[82] Practice Committee of the American Society for Reproductive Medicine. Electronic address Aao, Practice Committee of the American Society for Reproductive M (2016). Prevention and treatment of moderate and severe ovarian hyperstimulation syndrome: a guideline. Fertil Steril.

[83] Golan A, Herman A, Soffer Y, Bukovsky I, Ron-El R. Ultrasonic control without hormone determination for ovulation induction in in-vitro fertilization/embryo transfer with gonadotrophin-releasing hormone analogue and human menopausal gonadotrophin. Hum Reprod. 1994;9(9):1631–3. 10.1093/oxfordjournals.humrep.a138764 7836512

[84] Wiser A, Gonen O, Ghetler Y, Shavit T, Berkovitz A, Shulman A (2012). Monitoring stimulated cycles during in vitro fertilization treatment with ultrasound only--preliminary results. Gynecol Endocrinol.

[85] Klinkert ER, Broekmans FJ, Looman CW, Habbema JD, te Velde ER. Expected poor responders on the basis of an antral follicle count do not benefit from a higher starting dose of gonadotrophins in IVF treatment: a randomized controlled trial. Hum Reprod, 2005. 20(3):611–5. 10.1093/humrep/deh663 15591079

[86] Aboulghar MA, Mansour RT, Serour GI, Al-Inany HG, Amin YM, Aboulghar MM (2004). Increasing the dose of human menopausal gonadotrophins on day of GnRH antagonist administration: randomized controlled trial. Reprod Biomed Online.

[87] Aboulghar MA, Mansour RT, Serour GI, Rhodes CA, Amin YM (2000). Reduction of human menopausal gonadotropin dose before coasting prevents severe ovarian hyperstimulation syndrome with minimal cycle cancellation. J Assist Reprod Genet.

[88] La Marca A, Blockeel C, Bosch E, Fanchin R, Fatemi HM, Fauser BC (2018). Individualized FSH dosing improves safety and reduces iatrogenic poor response while maintaining live-birth rates. Hum Reprod.

[89] Sunkara SK, Polyzos NP (2018). OPTIMIST trial: optimistic evidence?. Hum Reprod.

[90] Delvigne A, Rozenberg S (2002). A qualitative systematic review of coasting, a procedure to avoid ovarian hyperstimulation syndrome in IVF patients. Hum Reprod Update.

[91] D'Angelo A, Amso NN, Hassan R. Coasting (withholding gonadotrophins) for preventing ovarian hyperstimulation syndrome. Cochrane Database Syst Rev. 2017;5(5):Cd002811. 10.1002/14651858.CD002811.pub4 PMC6481358 28535578

[92] Kamthane VP, Goswami SK, Ghosh S, Chattopadhyay R, Chakravarty BN (2004). Does coasting prevent OHSS without compromising pregnancy outcome?. Hum Reprod.

[93] Lukaszuk L, Liss J, Jakiel G (2011). Internal Coasting' for prevention of ovarian hyperstimulation syndrome (OHSS) in IVF/ICSI. Ginekol Pol.

