# Supplementary material for: Dose adjustment of follicle-stimulating hormone (FSH) during ovarian stimulation as part of medically-assisted reproduction in clinical studies: a systematic review covering 10 years (2007–2017)
Source: Reprod Biol Endocrinol. 2021 May 11;19:68. doi: 10.1186/s12958-021-00744-x (PMC8112039; doi:10.1186/s12958-021-00744-x)
Supplement: Supplementary file 1 — Additional file 1. [file 12958_2021_744_MOESM1_ESM.docx]

**Supplementary appendix**

**Supplementary table 1**. Data extraction forms for each study

| **Reference:** Allegra A, et al. Reprod Biomed Online 2017;34:429–38. | | |
| --- | --- | --- |
|  | **Study arm*** | |
|  | **Control** | **Nomogram** |
| **Population size, N** | 99 | 92 |
| **Dose data** |  |  |
| Fixed dose (yes/no) | No | No |
| Dose adjustments detailed (yes/no) | Yes | Yes |
| Gonadotropin starting dose (IU; Mean [SD]) | 182.6 (37.4) | 201.1 (28.4) |
| Day adjustments allowed from | 5 | 5 |
| Duration of gonadotropin treatment (Days; Mean [SD]) | 11.2 (1.5) | 10.8 (1.6) |
| Total gonadotropin dose (IU; Mean [SD]) | 1688.6 (484) | 1782.3 (536) |
| Unspecified dose adjustment reported |  |  |
| Unspecified dose adjustment, n | 72 | 56 |
| Unspecified dose adjustment, % (95% CI) | 72.7 (64.0, 81.5) | 60.9 (50.9, 70.8) |
| Dose increase reported |  |  |
| Dose increase, n | - | - |
| Dose increase, % (95% CI) | - | - |
| Dose decrease reported |  |  |
| Dose decrease, n | - | - |
| Dose decrease, % (95% CI) | - | - |
| **Studies with all data reported** |  |  |
| Unspecified dose adjustment reported |  |  |
| Unspecified dose adjustment, n | - | - |
| Unspecified dose adjustment, % (95% CI) | - | - |
| Dose increase reported |  |  |
| Dose increase, n | - | - |
| Dose increase, % (95% CI) | - | - |
| Dose decrease reported |  |  |
| Dose decrease, n | - | - |
| Dose decrease, % (95% CI) | - | - |
| **Dose adjustment in studies with biomarker starting doses** |  |  |
| Unspecified dose adjustment reported |  |  |
| Unspecified dose adjustment, n | 72 | 56 |
| Unspecified dose adjustment, % (95% CI) | 72.7 (64.0, 81.5) | 60.9 (50.9, 70.8) |
| Dose increase reported |  |  |
| Dose increase, n | - | - |
| Dose increase, % (95% CI) | - | - |
| Dose decrease reported |  |  |
| Dose decrease, n | - | - |
| Dose decrease, % (95% CI) | - | - |
| **FSH used** | GONAL-f | GONAL-f |
| **GnRH protocol (agonist or antagonist)** | Agonist | Agonist |
| **Biomarkers** |  |  |
| Was starting dose selected according to biomarkers? (yes/no) | Yes | Yes |
| Biomarkers used (if yes above) | Age | Age, AMH  and basal FSH |
| **Population** |  |  |
| Non-standard responders (e.g. POR, PCOS) investigated? (yes/no) | No | No |
| Population (if yes above) |  |  |
| *Only study arms using recombinant FSH were included in the data analysis.  CI, confidence interval; FSH, follicle stimulating hormone; PCOS, polycystic ovary syndrome; POR, poor ovarian response; SD, standard deviation | | |

| **Reference:** Buhler K, et al. Reprod Biol Endocrinol 2014;12:6. | |
| --- | --- |
| **Population size, N** | 2074* |
| **Dose data** |  |
| Fixed dose (yes/no) | No |
| Dose adjustments detailed (yes/no) | Yes |
| Gonadotropin starting dose (IU; Mean [SD]) | 183.1/91.5 (77.4/38.7) |
| Day adjustments allowed from | n/a |
| Duration of gonadotropin treatment (Days; Mean [SD]) | 10.8 (2.9) |
| Total gonadotropin dose (IU; Mean [SD]) | 1923.4/961.7 (1098.6/549.3) |
| Unspecified dose adjustment reported |  |
| Unspecified dose adjustment, n | - |
| Unspecified dose adjustment, % (95% CI) | - |
| Dose increase reported |  |
| Dose increase, n | 99 |
| Dose increase, % (95% CI) | 4.8 (3.9, 5.7) |
| Dose decrease reported |  |
| Dose decrease, n | 76 |
| Dose decrease, % (95% CI) | 3.7 (2.9, 4.5) |
| **Studies with all data reported** |  |
| Unspecified dose adjustment reported |  |
| Unspecified dose adjustment, n | - |
| Unspecified dose adjustment, % (95% CI) | - |
| Dose increase reported |  |
| Dose increase, n | 99 |
| Dose increase, % (95% CI) | 4.8 (3.9, 5.7) |
| Dose decrease reported |  |
| Dose decrease, n | 76 |
| Dose decrease, % (95% CI) | 3.7 (2.9, 4.5) |
| **Dose adjustment in studies with biomarker starting doses** |  |
| Unspecified dose adjustment reported |  |
| Unspecified dose adjustment, n | - |
| Unspecified dose adjustment, % (95% CI) | - |
| Dose increase reported |  |
| Dose increase, n | - |
| Dose increase, % (95% CI) | - |
| Dose decrease reported |  |
| Dose decrease, n | - |
| Dose decrease, % (95% CI) | - |
| **FSH used** | Pergoveris |
| **GnRH protocol (agonist or antagonist)** | n/a |
| **Biomarkers** |  |
| Was starting dose selected according to biomarkers? (yes/no) | No |
| Biomarkers used (if yes above) |  |
| **Population** |  |
| Non-standard responders (e.g. POR, PCOS) investigated? (yes/no) | No |
| Population (if yes above) |  |
| *2220 cycles assessed overall in study, with data on dose available in 2074 cycles.  CI, confidence interval; FSH, follicle stimulating hormone; n/a, not available; PCOS, polycystic ovary syndrome; POR, poor ovarian response; SD, standard deviation | |

| **Reference:** Magnusson Å, et al. Hum Reprod 2017;32:811–19. | | |
| --- | --- | --- |
|  | **Study arm*** | |
|  | **AMH** | **non-AMH** |
| **Population size, N** | 152 | 155 |
| **Dose data** |  |  |
| Fixed dose (yes/no) | No | No |
| Dose adjustments detailed (yes/no) | Yes | Yes |
| Gonadotropin starting dose (IU) | 75–300 | 75–300 |
| Day adjustments allowed from | 7 | 7 |
| Duration of gonadotropin treatment | n/a | n/a |
| Total gonadotropin dose (IU; Mean [SD]) | 1685 (997) | 1604 (701) |
| Unspecified dose adjustment reported |  |  |
| Unspecified dose adjustment, n | 83 | 81 |
| Unspecified dose adjustment, % (95% CI) | 54.6 (46.7, 62.5) | 52.3 (44.4, 60.1) |
| Dose increase reported |  |  |
| Dose increase, n | - | - |
| Dose increase, % (95% CI) | - | - |
| Dose decrease reported |  |  |
| Dose decrease, n | - | - |
| Dose decrease, % (95% CI) | - | - |
| **Studies with all data reported** |  |  |
| Unspecified dose adjustment reported |  |  |
| Unspecified dose adjustment, n | - | - |
| Unspecified dose adjustment, % (95% CI) | - | - |
| Dose increase reported |  |  |
| Dose increase, n | - | - |
| Dose increase, % (95% CI) | - | - |
| Dose decrease reported |  |  |
| Dose decrease, n | - | - |
| Dose decrease, % (95% CI) | - | - |
| **Dose adjustment in studies with biomarker starting doses** |  |  |
| Unspecified dose adjustment reported |  |  |
| Unspecified dose adjustment, n | 83 | 81 |
| Unspecified dose adjustment, % (95% CI) | 54.6 (46.7, 62.5) | 52.3 (44.4, 60.1) |
| Dose increase reported |  |  |
| Dose increase, n | - | - |
| Dose increase, % (95% CI) | - | - |
| Dose decrease reported |  |  |
| Dose decrease, n | - | - |
| Dose decrease, % (95% CI) | - | - |
| **FSH used** | rFSH | rFSH |
| **GnRH protocol (agonist or antagonist)** | Agonist | Agonist |
| **Biomarkers** |  |  |
| Was starting dose selected according to biomarkers? (yes/no) | Yes | Yes |
| Biomarkers used (if yes above) | Age, BMI, AFC and serum AMH | Age, BMI and AFC |
| **Population** |  |  |
| Non-standard responders (e.g. POR, PCOS) investigated? (yes/no) | No | No |
| Population (if yes above) |  |  |
| *Only study arms using recombinant FSH were included in the data analysis.  AFC, antral follicle count; AMH, anti-Müllerian hormone; BMI, body mass index; CI, confidence interval; FSH, follicle stimulating hormone; n/a, not available; PCOS, polycystic ovary syndrome; POR, poor ovarian response; rFSH, recombinant follicle stimulating hormone; SD, standard deviation | | |

| **Reference:** Espinós, JJ. Reprod Biomed Online 2017;35:417–24. | | |
| --- | --- | --- |
|  | **Study arm*** | |
|  | **Diet** | **Control** |
| **Population size, N** | 21 | 20 |
| **Dose data** |  |  |
| Fixed dose (yes/no) | No | No |
| Dose adjustments detailed (yes/no) | Yes | Yes |
| Gonadotropin starting dose (IU) | 150 (n=4) or 225 (n=17) | 150 (n=4) or 225 (n=16) |
| Day adjustments allowed from | n/a | n/a |
| Duration of gonadotropin treatment (Days; Mean [SD]) | 11.3 (2.2) | 11.2 (2.0) |
| Total gonadotropin dose (IU; Mean [SD]) | 2407 (513) | 2503 (875) |
| Unspecified dose adjustment reported |  | |
| Unspecified dose adjustment, n | 11 | |
| Unspecified dose adjustment, % (95% CI) | 26.8 (13.3, 40.4) | |
| Dose increase reported |  |  |
| Dose increase, n | - | - |
| Dose increase, % (95% CI) | - | - |
| Dose decrease reported |  |  |
| Dose decrease, n | - | - |
| Dose decrease, % (95% CI) | - | - |
| **Studies with all data reported** |  |  |
| Unspecified dose adjustment reported |  |  |
| Unspecified dose adjustment, n | - | - |
| Unspecified dose adjustment, % (95% CI) | - | - |
| Dose increase reported |  |  |
| Dose increase, n | - | - |
| Dose increase, % (95% CI) | - | - |
| Dose decrease reported |  |  |
| Dose decrease, n | - | - |
| Dose decrease, % (95% CI) | - | - |
| **Dose adjustment in studies with biomarker starting doses** |  |  |
| Unspecified dose adjustment reported |  | |
| Unspecified dose adjustment, n | 11 | |
| Unspecified dose adjustment, % (95% CI) | 26.8 (13.3, 40.4) | |
| Dose increase reported |  |  |
| Dose increase, n | - | - |
| Dose increase, % (95% CI) | - | - |
| Dose decrease reported |  |  |
| Dose decrease, n | - | - |
| Dose decrease, % (95% CI) | - | - |
| **FSH used** | GONAL-f | GONAL-f |
| **GnRH protocol (agonist or antagonist)** | Antagonist | Antagonist |
| **Biomarkers** |  |  |
| Was starting dose selected according to biomarkers? (yes/no) | Yes | Yes |
| Biomarkers used (if yes above) | AFC >13 | AFC >13 |
| **Population** |  |  |
| Non-standard responders (e.g. POR, PCOS) investigated? (yes/no) | Yes | Yes |
| Population (if yes above) | Obese women | Obese women |
| *Only study arms using recombinant FSH were included in the data analysis.  CI, confidence interval; FSH, follicle stimulating hormone; n/a, not available; PCOS, polycystic ovary syndrome; POR, poor ovarian response; SD, standard deviation | | |

| **Reference:** Nyboe Andersen A, et al. Fertil Steril 2017;107:387–96. | |
| --- | --- |
| **Population size, N** | 661 |
| **Dose data** |  |
| Fixed dose (yes/no) | No |
| Dose adjustments detailed (yes/no) | Yes |
| Gonadotropin starting dose (IU) | 150 |
| Day adjustments allowed from | 6 |
| Duration of gonadotropin treatment (Days; Mean [SD]) | 8.6 (1.7) |
| Total gonadotropin dose (μg; Mean [SD]) | 103.7 (33.6) |
| Unspecified dose adjustment reported |  |
| Unspecified dose adjustment, n | 243 |
| Unspecified dose adjustment, % (95% CI) | 36.8 (33.1, 40.4) |
| Dose increase reported |  |
| Dose increase, n | - |
| Dose increase, % (95% CI) | - |
| Dose decrease reported |  |
| Dose decrease, n | - |
| Dose decrease, % (95% CI) | - |
| **Studies with all data reported** |  |
| Unspecified dose adjustment reported |  |
| Unspecified dose adjustment, n | - |
| Unspecified dose adjustment, % (95% CI) | - |
| Dose increase reported |  |
| Dose increase, n | - |
| Dose increase, % (95% CI) | - |
| Dose decrease reported |  |
| Dose decrease, n | - |
| Dose decrease, % (95% CI) | - |
| **Dose adjustment in studies with biomarker starting doses** |  |
| Unspecified dose adjustment reported |  |
| Unspecified dose adjustment, n | - |
| Unspecified dose adjustment, % (95% CI) | - |
| Dose increase reported |  |
| Dose increase, n | - |
| Dose increase, % (95% CI) | - |
| Dose decrease reported |  |
| Dose decrease, n | - |
| Dose decrease, % (95% CI) | - |
| **FSH used** | GONAL-f |
| **GnRH protocol (agonist or antagonist)** | Antagonist |
| **Biomarkers** |  |
| Was starting dose selected according to biomarkers? (yes/no) | No |
| Biomarkers used (if yes above) |  |
| **Population** |  |
| Non-standard responders (e.g. POR, PCOS) investigated? (yes/no) | No |
| Population (if yes above) |  |
| CI, confidence interval; FSH, follicle stimulating hormone; PCOS, polycystic ovary syndrome; POR, poor ovarian response; SD, standard deviation | |

| **Reference:** Rettenbacher M, et al. Reprod Biomed Online 2015;30:504–13. | | |
| --- | --- | --- |
|  | **Study arm*** | |
|  | **Bemfola** | **GONAL-f** |
| **Population size, N** | 220 | 113 |
| **Dose data** |  |  |
| Fixed dose (yes/no) | No | No |
| Dose adjustments detailed (yes/no) | Yes | Yes |
| Gonadotropin starting dose (IU) | 150 | 150 |
| Day adjustments allowed from | 7 | 7 |
| Duration of gonadotropin treatment (Days; Mean [SD]) | 10.6 (1.91) | 10.7 (1.72) |
| Total gonadotropin dose (IU; Mean [SD]) | 1555.7 (293.00) | 1569.2 (259.20) |
| Unspecified dose adjustment reported |  |  |
| Unspecified dose adjustment, n | - | - |
| Unspecified dose adjustment, % (95% CI) | - | - |
| Dose increase reported |  |  |
| Dose increase, n | Not allowed | Not allowed |
| Dose increase, % (95% CI) |  |  |
| Dose decrease reported |  |  |
| Dose decrease, n | 38 | 16 |
| Dose decrease, % (95% CI) | 17.3 (12.3, 22.3) | 14.2 (7.7, 20.6) |
| **Studies with all data reported** |  |  |
| Unspecified dose adjustment reported |  |  |
| Unspecified dose adjustment, n | - | - |
| Unspecified dose adjustment, % (95% CI) | - | - |
| Dose increase reported |  |  |
| Dose increase, n | Not allowed | Not allowed |
| Dose increase, % (95% CI) |  |  |
| Dose decrease reported |  |  |
| Dose decrease, n | - | - |
| Dose decrease, % (95% CI) | - | - |
| **Dose adjustment in studies with biomarker starting doses** |  |  |
| Unspecified dose adjustment reported |  |  |
| Unspecified dose adjustment, n | - | - |
| Unspecified dose adjustment, % (95% CI) | - | - |
| Dose increase reported |  |  |
| Dose increase, n | Not allowed | Not allowed |
| Dose increase, % (95% CI) |  |  |
| Dose decrease reported |  |  |
| Dose decrease, n | - | - |
| Dose decrease, % (95% CI) | - | - |
| **FSH used** | Bemfola | GONAL-f |
| **GnRH protocol (agonist or antagonist)** | Agonist | Agonist |
| **Biomarkers** |  |  |
| Was starting dose selected according to biomarkers? (yes/no) | No | No |
| Biomarkers used (if yes above) |  |  |
| **Population** |  |  |
| Non-standard responders (e.g. POR, PCOS) investigated? (yes/no) | No | No |
| Population (if yes above) |  |  |
| *Only study arms using recombinant FSH were included in the data analysis.  CI, confidence interval; FSH, follicle stimulating hormone; PCOS, polycystic ovary syndrome; POR, poor ovarian response; SD, standard deviation | | |

| **Reference:** Strowitzki T, et al. Reprod Biol Endocrinol 2016;14:1. | | |
| --- | --- | --- |
|  | **Study arm*** | |
|  | **Ovaleap** | **GONAL-f** |
| **Population size, N** | 153 | 146 |
| **Dose data** |  |  |
| Fixed dose (yes/no) | No | No |
| Dose adjustments detailed (yes/no) | Yes | Yes |
| Gonadotropin starting dose (IU) | 150 | 150 |
| Day adjustments allowed from | 6 | 6 |
| Duration of gonadotropin treatment (Days; Mean [SD]) | 9.3 (1.8) | 9.7 (1.6) |
| Total gonadotropin dose (IU; Mean [SD]) | 1536 (496) | 1614 (485) |
| Unspecified dose adjustment reported |  |  |
| Unspecified dose adjustment, n | - | - |
| Unspecified dose adjustment, % (95% CI) | - | - |
| Dose increase reported |  |  |
| Dose increase, n | 55 | 63 |
| Dose increase, % (95% CI) | 35.9 (28.3, 43.6) | 43.2 (35.1, 51.2) |
| Dose decrease reported |  |  |
| Dose decrease, n | 23 | 22 |
| Dose decrease, % (95% CI) | 15.0 (9.4, 20.7) | 15.1 (9.3, 20.9) |
| **Studies with all data reported** |  |  |
| Unspecified dose adjustment reported |  |  |
| Unspecified dose adjustment, n | - | - |
| Unspecified dose adjustment, % (95% CI) | - | - |
| Dose increase reported |  |  |
| Dose increase, n | 55 | 63 |
| Dose increase, % (95% CI) | 35.9 (28.3, 43.6) | 43.2 (35.1, 51.2) |
| Dose decrease reported |  |  |
| Dose decrease, n | 23 | 22 |
| Dose decrease, % (95% CI) | 15.0 (9.4, 20.7) | 15.1 (9.3, 20.9) |
| **Dose adjustment in studies with biomarker starting doses** |  |  |
| Unspecified dose adjustment reported |  |  |
| Unspecified dose adjustment, n | - | - |
| Unspecified dose adjustment, % (95% CI) | - | - |
| Dose increase reported |  |  |
| Dose increase, n | - | - |
| Dose increase, % (95% CI) | - | - |
| Dose decrease reported |  |  |
| Dose decrease, n | - | - |
| Dose decrease, % (95% CI) | - | - |
| **FSH used** | **Ovaleap** | **GONAL-f** |
| **GnRH protocol (agonist or antagonist)** | Agonist | Agonist |
| **Biomarkers** |  |  |
| Was starting dose selected according to biomarkers? (yes/no) | No | No |
| Biomarkers used (if yes above) |  |  |
| **Population** |  |  |
| Non-standard responders (e.g. POR, PCOS) investigated? (yes/no) | No | No |
| Population (if yes above) |  |  |
| *Only study arms using recombinant FSH were included in the data analysis.  CI, confidence interval; FSH, follicle stimulating hormone; PCOS, polycystic ovary syndrome; POR, poor ovarian response; SD, standard deviation | | |

| **Reference:** Devroey P, et al. Fertil Steril 2012;97:561–71. | |
| --- | --- |
| **Population size, N** | 375 |
| **Dose data** |  |
| Fixed dose (yes/no) | No |
| Dose adjustments detailed (yes/no) | Yes |
| Gonadotropin starting dose (IU) | 150 |
| Day adjustments allowed from | 6 |
| Duration of gonadotropin treatment (Days; Mean [SD]) | 8.5 (1.3) |
| Total gonadotropin dose (IU; Mean [SD]) | 1353 (296) |
| Unspecified dose adjustment reported |  |
| Unspecified dose adjustment, n | - |
| Unspecified dose adjustment, % (95% CI) | - |
| Dose increase reported |  |
| Dose increase, n | 93 |
| Dose increase, % (95% CI) | 24.8 (20.4, 29.2) |
| Dose decrease reported | - |
| Dose decrease, n | 8 |
| Dose decrease, % (95% CI) | 2.1 (0.7, 3.6) |
| **Studies with all data reported** |  |
| Unspecified dose adjustment reported |  |
| Unspecified dose adjustment, n | - |
| Unspecified dose adjustment, % (95% CI) | - |
| Dose increase reported | - |
| Dose increase, n | - |
| Dose increase, % (95% CI) | - |
| Dose decrease reported |  |
| Dose decrease, n | - |
| Dose decrease, % (95% CI) | - |
| **Dose adjustment in studies with biomarker starting doses** |  |
| Unspecified dose adjustment reported |  |
| Unspecified dose adjustment, n | - |
| Unspecified dose adjustment, % (95% CI) | - |
| Dose increase reported |  |
| Dose increase, n | - |
| Dose increase, % (95% CI) | - |
| Dose decrease reported |  |
| Dose decrease, n | - |
| Dose decrease, % (95% CI) | - |
| **FSH used** | Puregon |
| **GnRH protocol (agonist or antagonist)** | Antagonist |
| **Biomarkers** |  |
| Was starting dose selected according to biomarkers? (yes/no) | No |
| Biomarkers used (if yes above) |  |
| **Population** |  |
| Non-standard responders (e.g. POR, PCOS) investigated? (yes/no) | No |
| Population (if yes above) |  |
| CI, confidence interval; FSH, follicle stimulating hormone; PCOS, polycystic ovary syndrome; POR, poor ovarian response; SD, standard deviation | |

| **Reference:** Durnerin CI, et al. Hum Reprod 2008;23:421–6. | | |
| --- | --- | --- |
|  | **Study arm*** | |
|  | **Control** | **r-hLH pretreat** |
| **Population size, N** | 49 | 53 |
| **Dose data** |  |  |
| Fixed dose (yes/no) | No | No |
| Dose adjustments detailed (yes/no) | No | No |
| Gonadotropin starting dose (IU) | 150 | 150 |
| Day adjustments allowed from | 8 | 8 |
| Duration of gonadotropin treatment (Days; Mean [SD]) | 12.1 | 12.5 |
| Total gonadotropin dose (IU; Mean [SD]) | 2221 | 2282 |
| Unspecified dose adjustment reported |  |  |
| Unspecified dose adjustment, n | - | - |
| Unspecified dose adjustment, % (95% CI) | - | - |
| Dose increase reported |  |  |
| Dose increase, n | 27 | 29 |
| Dose increase, % (95% CI) | 55.1 (41.2, 69.0) | 54.7 (41.3, 68.1) |
| Dose decrease reported |  |  |
| Dose decrease, n | 1 | 2 |
| Dose decrease, % (95% CI) | 2.0 (−1.9, 6.0) | 3.8 (−1.4, 8.9) |
| **Studies with all data reported** |  |  |
| Unspecified dose adjustment reported |  |  |
| Unspecified dose adjustment, n | - | - |
| Unspecified dose adjustment, % (95% CI) | - | - |
| Dose increase reported |  |  |
| Dose increase, n | - | - |
| Dose increase, % (95% CI) | - | - |
| Dose decrease reported |  |  |
| Dose decrease, n | - | - |
| Dose decrease, % (95% CI) | - | - |
| **Dose adjustment in studies with biomarker starting doses** |  |  |
| Unspecified dose adjustment reported |  |  |
| Unspecified dose adjustment, n | - | - |
| Unspecified dose adjustment, % (95% CI) | - | - |
| Dose increase reported |  |  |
| Dose increase, n | - | - |
| Dose increase, % (95% CI) | - | - |
| Dose decrease reported |  |  |
| Dose decrease, n | - | - |
| Dose decrease, % (95% CI) | - | - |
| **FSH used** | GONAL-f | GONAL-f |
| **GnRH protocol (agonist or antagonist)** | Agonist | Agonist |
| **Biomarkers** |  |  |
| Was starting dose selected according to biomarkers? (yes/no) | No | No |
| Biomarkers used (if yes above) |  |  |
| **Population** |  |  |
| Non-standard responders (e.g. POR, PCOS) investigated? (yes/no) | No | No |
| Population (if yes above) |  |  |
| *Only study arms using recombinant FSH were included in the data analysis.  CI, confidence interval; FSH, follicle stimulating hormone; PCOS, polycystic ovary syndrome; POR, poor ovarian response; SD, standard deviation | | |

| **Reference:** Esteves SC, et al. Reprod Biol Endocrinol 2009;7:111. | |
| --- | --- |
|  | **Study arm*** |
|  | r-hFSH |
| **Population size, N** | 236 |
| **Dose data** |  |
| Fixed dose (yes/no) | No |
| Dose adjustments detailed (yes/no) | Yes |
| Gonadotropin starting dose (IU) | 150–375 |
| Day adjustments allowed from | 6 |
| Duration of gonadotropin treatment (Days; Mean [SD]) | 10.1 (1.0) |
| Total gonadotropin dose (IU; Mean [SD]) | n/a |
| Unspecified dose adjustment reported |  |
| Unspecified dose adjustment, n | - |
| Unspecified dose adjustment, % (95% CI) | - |
| Dose increase reported |  |
| Dose increase, n | - |
| Dose increase, % (95% CI) | - |
| Dose decrease reported |  |
| Dose decrease, n | 126 |
| Dose decrease, % (95% CI) | 53.4 (47.0, 59.8) |
| **Studies with all data reported** |  |
| Unspecified dose adjustment reported |  |
| Unspecified dose adjustment, n | - |
| Unspecified dose adjustment, % (95% CI) | - |
| Dose increase reported |  |
| Dose increase, n | - |
| Dose increase, % (95% CI) | - |
| Dose decrease reported |  |
| Dose decrease, n | 126 |
| Dose decrease, % (95% CI) | 53.4 (47.0, 59.8) |
| **Dose adjustment in studies with biomarker starting doses** |  |
| Unspecified dose adjustment reported |  |
| Unspecified dose adjustment, n | - |
| Unspecified dose adjustment, % (95% CI) | - |
| Dose increase reported |  |
| Dose increase, n | - |
| Dose increase, % (95% CI) | - |
| Dose decrease reported |  |
| Dose decrease, n | - |
| Dose decrease, % (95% CI) | - |
| **FSH used** | GONAL-f |
| **GnRH protocol (agonist or antagonist)** | Agonist |
| **Biomarkers** |  |
| Was starting dose selected according to biomarkers? (yes/no) | Yes |
| Biomarkers used (if yes above) | Age, BMI, serum FSH on day 2 or 3 of the menstrual cycle, baseline ovarian volume on TVUS, and number of pre-antral follicles between days 2 and 3 of the menstrual cycle after pituitary down-regulation |
| **Population** |  |
| Non-standard responders (e.g. POR, PCOS) investigated? (yes/no) | No |
| Population (if yes above) |  |
| *Only study arms using recombinant FSH were included in the data analysis.  BMI, body mass index, CI, confidence interval; FSH, follicle stimulating hormone; n/a, not available; PCOS, polycystic ovary syndrome; POR, poor ovarian response; SD, standard deviation; TVUS, transvaginal ultrasound | |

| **Reference:** Devroey P, et al. Hum Reprod 2009;24:3063–72. | |
| --- | --- |
| **Population size, N** | 750* |
| **Dose data** |  |
| Fixed dose (yes/no) | No |
| Dose adjustments detailed (yes/no) | Yes |
| Gonadotropin starting dose (IU) | 200 |
| Day adjustments allowed from | 6 |
| Duration of gonadotropin treatment (Days; Median) | 9 |
| Total gonadotropin dose | n/a |
| Unspecified dose adjustment reported |  |
| Unspecified dose adjustment, n | - |
| Unspecified dose adjustment, % (95% CI) | - |
| Dose increase reported |  |
| Dose increase, n | - |
| Dose increase, % (95% CI) | - |
| Dose decrease reported |  |
| Dose decrease, n | 62 |
| Dose decrease, % (95% CI) | 8.4 (6.4, 10.4) |
| **Studies with all data reported** |  |
| Unspecified dose adjustment reported |  |
| Unspecified dose adjustment, n | - |
| Unspecified dose adjustment, % (95% CI) | - |
| Dose increase reported |  |
| Dose increase, n | - |
| Dose increase, % (95% CI) | - |
| Dose decrease reported |  |
| Dose decrease, n | - |
| Dose decrease, % (95% CI) | - |
| **Dose adjustment in studies with biomarker starting doses** |  |
| Unspecified dose adjustment reported |  |
| Unspecified dose adjustment, n | - |
| Unspecified dose adjustment, % (95% CI) | - |
| Dose increase reported |  |
| Dose increase, n | - |
| Dose increase, % (95% CI) | - |
| Dose decrease reported |  |
| Dose decrease, n | - |
| Dose decrease, % (95% CI) | - |
| **FSH used** | Puregon |
| **GnRH protocol (agonist or antagonist)** | Antagonist |
| **Biomarkers** |  |
| Was starting dose selected according to biomarkers? (yes/no) | No |
| Biomarkers used (if yes above) |  |
| **Population** |  |
| Non-standard responders (e.g. POR, PCOS) investigated? (yes/no) | No |
| Population (if yes above) |  |
| *N=750 patients in the treated population, of which N=741 patients were eligible for a potential dose decrease on Days 6 or 7. CI, confidence interval; FSH, follicle stimulating hormone; n/a, not available; PCOS, polycystic ovary syndrome; POR, poor ovarian response; SD, standard deviation | |

| **Reference:** Freiesleben NL, et al. Reprod Biomed Online 2008;17:632–41. | |
| --- | --- |
| **Population size, N** | 159 |
| **Dose data** |  |
| Fixed dose (yes/no) | No |
| Dose adjustments detailed (yes/no) | Yes |
| Gonadotropin starting dose (IU) | 75 |
| Day adjustments allowed from | 6 |
| Duration of gonadotropin treatment (Days; Mean [SD]) | 8.1 (2.3) |
| Total gonadotropin dose (IU; Mean [SD]) | 632 (338) |
| Unspecified dose adjustment reported |  |
| Unspecified dose adjustment, n | 70 |
| Unspecified dose adjustment, % (95% CI) | 44.0 (36.3, 51.7) |
| Dose increase reported |  |
| Dose increase, n | - |
| Dose increase, % (95% CI) | - |
| Dose decrease reported |  |
| Dose decrease, n | - |
| Dose decrease, % (95% CI) | - |
| **Studies with all data reported** |  |
| Unspecified dose adjustment reported |  |
| Unspecified dose adjustment, n | - |
| Unspecified dose adjustment, % (95% CI) | - |
| Dose increase reported |  |
| Dose increase, n | - |
| Dose increase, % (95% CI) | - |
| Dose decrease reported |  |
| Dose decrease, n | - |
| Dose decrease, % (95% CI) | - |
| **Dose adjustment in studies with biomarker starting doses** |  |
| Unspecified dose adjustment reported |  |
| Unspecified dose adjustment, n | - |
| Unspecified dose adjustment, % (95% CI) | - |
| Dose increase reported |  |
| Dose increase, n | - |
| Dose increase, % (95% CI) | - |
| Dose decrease reported |  |
| Dose decrease, n | - |
| Dose decrease, % (95% CI) | - |
| **FSH used** | Puregon |
| **GnRH protocol (agonist or antagonist)** | Antagonist |
| **Biomarkers** |  |
| Was starting dose selected according to biomarkers? (yes/no) | No |
| Biomarkers used (if yes above) |  |
| **Population** |  |
| Non-standard responders (e.g. POR, PCOS) investigated? (yes/no) | No |
| Population (if yes above) |  |
| CI, confidence interval; FSH, follicle stimulating hormone; PCOS, polycystic ovary syndrome; POR, poor ovarian response; SD, standard deviation | |

| **Reference:** Kyrou D, et al. Hum Reprod 2009;24:2902–9. | |
| --- | --- |
| **Population size, N** | 230 |
| **Dose data** |  |
| Fixed dose (yes/no) | No |
| Dose adjustments detailed (yes/no) | Yes |
| Gonadotropin starting dose (IU) | 200 |
| Day adjustments allowed from | 11 |
| Duration of gonadotropin treatment | n/a |
| Total gonadotropin dose | n/a |
| Unspecified dose adjustment reported |  |
| Unspecified dose adjustment, n | - |
| Unspecified dose adjustment, % (95% CI) | - |
| Dose increase reported |  |
| Dose increase, n | 7 |
| Dose increase, % (95% CI) | 3.0 (0.8, 5.3) |
| Dose decrease reported |  |
| Dose decrease, n | 16 |
| Dose decrease, % (95% CI) | 7.0 (3.7, 10.2) |
| **Studies with all data reported** |  |
| Unspecified dose adjustment reported |  |
| Unspecified dose adjustment, n | - |
| Unspecified dose adjustment, % (95% CI) | - |
| Dose increase reported |  |
| Dose increase, n | - |
| Dose increase, % (95% CI) | - |
| Dose decrease reported |  |
| Dose decrease, n | - |
| Dose decrease, % (95% CI) | - |
| **Dose adjustment in studies with biomarker starting doses** |  |
| Unspecified dose adjustment reported |  |
| Unspecified dose adjustment, n | - |
| Unspecified dose adjustment, % (95% CI) | - |
| Dose increase reported |  |
| Dose increase, n | - |
| Dose increase, % (95% CI) | - |
| Dose decrease reported |  |
| Dose decrease, n | - |
| Dose decrease, % (95% CI) | - |
| **FSH used** | Puregon |
| **GnRH protocol (agonist or antagonist)** | Antagonist |
| **Biomarkers** |  |
| Was starting dose selected according to biomarkers? (yes/no) | No |
| Biomarkers used (if yes above) |  |
| **Population** |  |
| Non-standard responders (e.g. POR, PCOS) investigated? (yes/no) | No |
| Population (if yes above) |  |
| CI, confidence interval; FSH, follicle stimulating hormone; n/a, not available; PCOS, polycystic ovary syndrome; POR, poor ovarian response; SD, standard deviation | |

| **Reference:** Lossl K, et al. Hum Reprod 2008;23:1820–9. | | |
| --- | --- | --- |
|  | **Study arm*** | |
|  | **Androgen priming** | **Control** |
| **Population size, N** | 53 | 50 |
| **Dose data** |  |  |
| Fixed dose (yes/no) | No | No |
| Dose adjustments detailed (yes/no) | Yes | Yes |
| Gonadotropin starting dose (IU) | 150 | 150 |
| Day adjustments allowed from | 6 | 6 |
| Duration of gonadotropin treatment (Days; Median [IQR]) | 10 (9, 10) | 8 (8, 9) |
| Total gonadotropin dose (IU; Median [IQR]) | 1650 (1575, 1875) | 1425 (1275, 1575) |
| Unspecified dose adjustment reported |  |  |
| Unspecified dose adjustment, n | - | - |
| Unspecified dose adjustment, % (95% CI) | - | - |
| Dose increase reported |  |  |
| Dose increase, n | 31 | 26 |
| Dose increase, % (95% CI) | 58.5 (45.2, 71.8) | 52.0 (38.2, 65.8) |
| Dose decrease reported |  |  |
| Dose decrease, n | 1 | 1 |
| Dose decrease, % (95% CI) | 1.9 (−1.8, 5.5) | 2.0 (−1.9, 5.9) |
| **Studies with all data reported** |  |  |
| Unspecified dose adjustment reported |  |  |
| Unspecified dose adjustment, n | - | - |
| Unspecified dose adjustment, % (95% CI) | - | - |
| Dose increase reported |  |  |
| Dose increase, n | - | - |
| Dose increase, % (95% CI) | - | - |
| Dose decrease reported |  |  |
| Dose decrease, n | - | - |
| Dose decrease, % (95% CI) | - | - |
| **Dose adjustment in studies with biomarker starting doses** |  |  |
| Unspecified dose adjustment reported |  |  |
| Unspecified dose adjustment, n | - | - |
| Unspecified dose adjustment, % (95% CI) | - | - |
| Dose increase reported |  |  |
| Dose increase, n | - | - |
| Dose increase, % (95% CI) | - | - |
| Dose decrease reported |  |  |
| Dose decrease, n | - | - |
| Dose decrease, % (95% CI) | - | - |
| **FSH used** | GONAL-f | GONAL-f |
| **GnRH protocol (agonist or antagonist)** | Antagonist | Antagonist |
| **Biomarkers** |  |  |
| Was starting dose selected according to biomarkers? (yes/no) | No | No |
| Biomarkers used (if yes above) |  |  |
| **Population** |  |  |
| Non-standard responders (e.g. POR, PCOS) investigated? (yes/no) | No | No |
| Population (if yes above) |  |  |
| *Only study arms using recombinant FSH were included in the data analysis.  CI, confidence interval; FSH, follicle stimulating hormone; IQR, interquartile range; PCOS, polycystic ovary syndrome; POR, poor ovarian response; SD, standard deviation | | |

| **Reference:** Nakhuda GS, et al. Reprod Biomed Online. 2010;20:42–7. | |
| --- | --- |
| **Population size, N** | 104 |
| **Dose data** |  |
| Fixed dose (yes/no) | No |
| Dose adjustments detailed (yes/no) | Yes |
| Gonadotropin starting dose (IU) | 150 r-hFSH + 75 hMG |
| Day adjustments allowed from | 5 |
| Duration of gonadotropin treatment | n/a |
| Total gonadotropin dose | n/a |
| Unspecified dose adjustment reported |  |
| Unspecified dose adjustment, n | - |
| Unspecified dose adjustment, % (95% CI) | - |
| Dose increase reported |  |
| Dose increase, n | 11 |
| Dose increase, % (95% CI) | 10.6 (4.7, 16.5) |
| Dose decrease reported |  |
| Dose decrease, n | 37 |
| Dose decrease, % (95% CI) | 35.6 (26.4, 44.8) |
| **Studies with all data reported** |  |
| Unspecified dose adjustment reported |  |
| Unspecified dose adjustment, n | - |
| Unspecified dose adjustment, % (95% CI) | - |
| Dose increase reported |  |
| Dose increase, n | 11 |
| Dose increase, % (95% CI) | 10.6 (4.7, 16.5) |
| Dose decrease reported |  |
| Dose decrease, n | 37 |
| Dose decrease, % (95% CI) | 35.6 (26.4, 44.8) |
| **Dose adjustment in studies with biomarker starting doses** |  |
| Unspecified dose adjustment reported |  |
| Unspecified dose adjustment, n | - |
| Unspecified dose adjustment, % (95% CI) | - |
| Dose increase reported |  |
| Dose increase, n | - |
| Dose increase, % (95% CI) | - |
| Dose decrease reported |  |
| Dose decrease, n | - |
| Dose decrease, % (95% CI) | - |
| **FSH used** | GONAL-f + Repronex |
| **GnRH protocol (agonist or antagonist)** | n/a |
| **Biomarkers** |  |
| Was starting dose selected according to biomarkers? (yes/no) | No |
| Biomarkers used (if yes above) |  |
| **Population** |  |
| Non-standard responders (e.g. POR, PCOS) investigated? (yes/no) | Yes |
| Population (if yes above) | Oocyte donors |
| CI, confidence interval; FSH, follicle stimulating hormone; n/a, not available; PCOS, polycystic ovary syndrome; POR, poor ovarian response; SD, standard deviation | |

| **Reference:** NyboeAndersen, et al. Hum Reprod 2008;23:427–34. | | |
| --- | --- | --- |
|  | **Study arm*** | |
|  | **r-hFSH** | **r-hFSH + r-hLH** |
| **Population size, N** | 261 | 265 |
| **Dose data** |  |  |
| Fixed dose (yes/no) | No | No |
| Dose adjustments detailed (yes/no) | Yes | Yes |
| Gonadotropin starting dose (IU) | 150–225 | 150–225 |
| Day adjustments allowed from | 7 | 7 |
| Duration of gonadotropin treatment (Days; Mean [SD]) | 11.2 (1.8) | 11.2 (2.1) |
| Total gonadotropin dose (IU; Mean [SD]) | 2131.6 (626.5) | 2139.3 (757.6) |
| Unspecified dose adjustment reported |  |  |
| Unspecified dose adjustment, n | - | - |
| Unspecified dose adjustment, % (95% CI) | - | - |
| Dose increase reported |  |  |
| Dose increase, n | 135 | 129 |
| Dose increase, % (95% CI) | 51.7 (45.7, 57.8) | 48.7 (42.7, 54.7) |
| Dose decrease reported |  |  |
| Dose decrease, n | 30 | 30 |
| Dose decrease, % (95% CI) | 11.5 (7.6, 15.4) | 11.3 (7.5, 15.1) |
| **Studies with all data reported** |  |  |
| Unspecified dose adjustment reported |  |  |
| Unspecified dose adjustment, n | - | - |
| Unspecified dose adjustment, % (95% CI) | - | - |
| Dose increase reported |  |  |
| Dose increase, n | 135 | 129 |
| Dose increase, % (95% CI) | 51.7 (45.7, 57.8) | 48.7 (42.7, 54.7) |
| Dose decrease reported |  |  |
| Dose decrease, n | 30 | 30 |
| Dose decrease, % (95% CI) | 11.5 (7.6, 15.4) | 11.3 (7.5, 15.1) |
| **Dose adjustment in studies with biomarker starting doses** |  |  |
| Unspecified dose adjustment reported |  |  |
| Unspecified dose adjustment, n | - | - |
| Unspecified dose adjustment, % (95% CI) | - | - |
| Dose increase reported |  |  |
| Dose increase, n | 135 | 129 |
| Dose increase, % (95% CI) | 51.7 (45.7, 57.8) | 48.7 (42.7, 54.7) |
| Dose decrease reported |  |  |
| Dose decrease, n | 30 | 30 |
| Dose decrease, % (95% CI) | 11.5 (7.6, 15.4) | 11.3 (7.5, 15.1) |
| **FSH used** | GONAL-f | Luveris |
| **GnRH protocol (agonist or antagonist)** | Agonist | Agonist |
| **Biomarkers** |  |  |
| Was starting dose selected according to biomarkers? (yes/no) | Yes | Yes |
| Biomarkers used (if yes above) | Age | Age |
| **Population** |  |  |
| Non-standard responders (e.g. POR, PCOS) investigated? (yes/no) | No | No |
| Population (if yes above) |  |  |
| *Only study arms using recombinant FSH were included in the data analysis.  CI, confidence interval; FSH, follicle stimulating hormone; PCOS, polycystic ovary syndrome; POR, poor ovarian response; SD, standard deviation | | |

| **Reference:** Requena A, et al. Hum Fertil (Camb) 2010;13:41–9. | | |
| --- | --- | --- |
|  | **Study arm*** | |
|  | **r-hFSH + hp-hMG** | **r-hFSH** |
| **Population size, N** | 46 | 46 |
| **Dose data** |  |  |
| Fixed dose (yes/no) | No | No |
| Dose adjustments detailed (yes/no) | Yes | Yes |
| Gonadotropin starting dose (IU) | 225 | 225 |
| Day adjustments allowed from | 4 | 4 |
| Duration of gonadotropin treatment | n/a | n/a |
| Total gonadotropin dose (IU; Mean [SD]) | 1406.33 (110.97) | 2110.85 (841.69) |
| Unspecified dose adjustment reported |  |  |
| Unspecified dose adjustment, n | - | - |
| Unspecified dose adjustment, % (95% CI) | - | - |
| Dose increase reported |  |  |
| Dose increase, n | 15 | 15 |
| Dose increase, % (95% CI) | 32.6 (19.1, 46.2) | 32.6 (19.1, 46.2) |
| Dose decrease reported |  |  |
| Dose decrease, n | - | - |
| Dose decrease, % (95% CI) | - | - |
| **Studies with all data reported** |  |  |
| Unspecified dose adjustment reported |  |  |
| Unspecified dose adjustment, n | - | - |
| Unspecified dose adjustment, % (95% CI) | - | - |
| Dose increase reported |  |  |
| Dose increase, n | - | - |
| Dose increase, % (95% CI) | - | - |
| Dose decrease reported |  |  |
| Dose decrease, n | - | - |
| Dose decrease, % (95% CI) | - | - |
| **Dose adjustment in studies with biomarker starting doses** |  |  |
| Unspecified dose adjustment reported |  |  |
| Unspecified dose adjustment, n | - | - |
| Unspecified dose adjustment, % (95% CI) | - | - |
| Dose increase reported |  |  |
| Dose increase, n | - | - |
| Dose increase, % (95% CI) | - | - |
| Dose decrease reported |  |  |
| Dose decrease, n | - | - |
| Dose decrease, % (95% CI) | - | - |
| **FSH used** | Menopur + GONAL-f | GONAL-f |
| **GnRH protocol (agonist or antagonist)** | Agonist | Agonist |

**Antagonist**

| **Biomarkers** |  |  |
| --- | --- | --- |
| Was starting dose selected according to biomarkers? (yes/no) | No | No |
| Biomarkers used (if yes above) |  |  |
| **Population** |  |  |
| Non-standard responders (e.g. POR, PCOS) investigated? (yes/no) | No | No |
| Population (if yes above) |  |  |
| *Only study arms using recombinant FSH were included in the data analysis.  CI, confidence interval; FSH, follicle stimulating hormone; n/a, not available; PCOS, polycystic ovary syndrome; POR, poor ovarian response; SD, standard deviation | | |

| **Reference:** Yovich J, et al. Reprod Biomed Online 2012;24:281–92. | |
| --- | --- |
|  | **Study arm*** |
|  | **FSH dose <100 IU** |
| **Population size, N** | 47 |
| **Dose data** |  |
| Fixed dose (yes/no) | No |
| Dose adjustments detailed (yes/no) | Yes |
| Gonadotropin starting dose (IU) | <100 |
| Day adjustments allowed from | 8 |
| Duration of gonadotropin treatment (Days; Median [IQR]) | n/a |
| Total gonadotropin dose (IU; Mean [SD]) | n/a |
| Unspecified dose adjustment reported |  |
| Unspecified dose adjustment, n | - |
| Unspecified dose adjustment, % (95% CI) | - |
| Dose increase reported |  |
| Dose increase, n | 25 |
| Dose increase, % (95% CI) | 53.2 (38.9, 67.5) |
| Dose decrease reported |  |
| Dose decrease, n | - |
| Dose decrease, % (95% CI) | - |
| **Studies with all data reported** |  |
| Unspecified dose adjustment reported |  |
| Unspecified dose adjustment, n | - |
| Unspecified dose adjustment, % (95% CI) | - |
| Dose increase reported |  |
| Dose increase, n | 25 |
| Dose increase, % (95% CI) | 53.2 (38.9, 67.5) |
| Dose decrease reported |  |
| Dose decrease, n | - |
| Dose decrease, % (95% CI) | - |
| **Dose adjustment in studies with biomarker starting doses** |  |
| Unspecified dose adjustment reported |  |
| Unspecified dose adjustment, n | - |
| Unspecified dose adjustment, % (95% CI) | - |
| Dose increase reported |  |
| Dose increase, n | 25 |
| Dose increase, % (95% CI) | 53.2 (38.9, 67.5) |
| Dose decrease reported |  |
| Dose decrease, n | - |
| Dose decrease, % (95% CI) | - |
| **FSH used** | Puregon or GONAL-f |
| **GnRH protocol (agonist or antagonist)** | Both |
| **Biomarkers** |  |
| Was starting dose selected according to biomarkers? (yes/no) | Yes |
| Biomarkers used (if yes above) | Day-2 FSH, AMH, AFC, BMI, age and smoking status? |
| **Population** |  |
| Non-standard responders (e.g. POR, PCOS) investigated? (yes/no) | No |
| Population (if yes above) |  |
| *Only study arms using recombinant FSH were included in the data analysis.  AFC, antral follicle count; AMH, anti-Müllerian hormone; BMI, body mass index; CI, confidence interval; FSH, follicle stimulating hormone; IQR, interquartile range; n/a, not available; PCOS, polycystic ovary syndrome; POR, poor ovarian response; SD, standard deviation | |

**Supplementary table 2**. Data available on baseline characteristics

| **Study** | **Study arm** | **Age** | **BMI** | **AMH** | **AFC** | **D3 FSH** |
| --- | --- | --- | --- | --- | --- | --- |
| **Allegra 2017** | **Control (N=99)** | 34.4 (3.9) | 22.7 (2.2) | 2.5 (0.9) | 10.8 (4.9) | 7.9 (5.3) |
|  | **Nomogram* (N=92)** | 33.5 (4.3) | 22.4 (2.3) | 2.4 (1.0) | 11.7 (5.5) | 7.1 (2.5) |
| **Buhler 2014** | **(N=2074^†^)** | 36.5 (4.00) | 23.1 (3.92) | 1.7 (2.06) | 5.3 (4.29) | 10.4 (11.31) |
| **Magnusson 2017** | **AMH (N=152)** | 32.3 (4.0) | 23.6 (3.7) | 4.03 (3.53) | 21.6 (12.0) | - |
|  | **Non-AMH (N=155)** | 32.3 (3.8) | 23.5 (3.6) | - | 21.3 (11.3) | - |
| **Espinós 2017** | **Diet (N=21)** | 32.0 (3.2) | 34.6 (3.0) | - | 17.6 (6.6) | - |
|  | **Control (N=20)** | 32.9 (3.9) | 34.0 (4.1) | - | 17.3 (6.5) | - |
| **Nyboe Andersen 2017** | **r-hFSH (N=661)** | 33.2 (3.9) | 23.3 (3.3) | 16.0 (9.1–25.5)* | 14.4 (6.8) | 7.7 (6.5–9.4)* |
| **Rettenbacher 2015** | **Bemfola (N=220)** | 31.8 (4.03) | 22.7 (2.88) | - | 15.1 (3.77) | 6.9 (1.50) |
|  | **GONAL-f (N=113)** | 32.1 (3.76) | 22.4 (2.56) | - | 15.3 (3.83) | 6.9 (1.56) |
| **Strowitzki 2016** | **Ovaleap (N=153)** | 31.6 (3.1) | 22.8 (2.9) | - | - | 7.0 (1.6) |
|  | **GONAL-f (N=146)** | 31.7 (3.2) | 22.6 (2.9) | - | - | 7.3 (2.3) |
| **Devroey 2012** | **r-hFSH (N=375)** | 30.4 (2.6) | 21.9 (2.0) | 27 (20) | 15.7 (5.8) | 7.4 (2.4) |
| **Durnerin 2008** | **Control (N=49)** | 32.6 (31.5, 33.4)^‡^ | 23.9 (23.0, 24.8)^‡^ | 20.0 (2.9) ^§^ | - |  |
|  | **r-hLH pretreat (N=53)** | 33.3 (31.0, 35.7)^‡^ | 23.6 (22.8, 24.4)^‡^ | 23.3 (2.7) ^§^ | - |  |
| **Esteves 2009** | **r-hFSH (N=236)** | 34.3 (4.8) | - | - | - | 6.5 (2.8) |
| **Devroey 2009** | **r-hFSH (N=750)** | 31.5 (3.2) | 24.8 (2.7) | - | - | - |
| **Freiesleben 2008** | **r-hFSH (N=159)** | 32.5 (3.8) | 22.7 (3.4) | - | 18.3 (9.4) | 7.9 (2.7) |
| **Kyrou 2009** | **r-hFSH (N=230)** | 32.9 (3.7) | - | - | - | - |
| **Lossl 2008**^#^ | **Androgen priming (N=53)** | 33 (26,29) | 21 (19,27) | - | 15 (9,32) | 6.9 (4.5, 12.2) |
|  | **Control (N=50)** | 32 (25,28) | 22 (19,30) | - | 17 (10,31) | 7.3 (4.9, 12.0) |
| **Nakhuda 2010**^§^ | **(N=104)** | 25.5 (0.29) | 21.03 (0.17) | 3.17 (0.20) | - | - |
| **Nyboe Andersen 2008** | **r-hFSH (N=261)** | 31.80 (3.98) | - | - | - | - |
|  | **r-hFSH + r-hLH (N=265)** | 31.72 (3.87) | - | - | - | - |
| **Requena 2010** | **r-hFSH + HP-hMG (N=46)** | 33.21 (3.47) | 22.76 (2.35) | - | - | 6.62 (1.54) |
|  | **r-hFSH (N=46)** | 31.57 (3.28) | 22.76 (2.56) | - | - | - |
| **Yovich 2012** | **FSH dose <100 IU (N=47)** | - | - | - | - | - |
| Data presented as mean (SD) unless otherwise stated. *Data presented as median (IQR); ^†^2220 cycles assessed overall in study, with data on dose available in 2074 cycles; ^‡^Data presented as mean (confidence limits); ^§^Data presented as mean (SEM); ^#^Data presented as mean (5^th^ centile, 95th centile).  AMH, anti-Müllerian hormone, FSH, follicle stimulating hormone; HP-hMG, highly purified human menopausal gonadotropin; IQR, interquartile range; r-hLH , recombinant human luteinizing hormone; r-hFSH, recombinant human follicle stimulating hormone; SD, standard deviation; SEM, standard error of the mean. | | | | | | |

|  |
| --- |

**Supplementary table 3**. Biomarkers measured before treatment and the type of FSH used.

| **Study** | **Study arm** | **Biomarkers** | **FSH used** |
| --- | --- | --- | --- |
| **Allegra 2017** | **Control (N=99)** | Age | GONAL-f® |
|  | **Nomogram* (N=92)** | AMH, Basal FSH, Female age | GONAL-f® |
| **Buhler 2014** | **(N=2074)** | N/A | Pergoveris® |
| **Magnusson 2017** | **AMH (N=152)** | AFC, Age, BMI, Serum AMH | GONAL-f® or Puregon® |
|  | **Non-AMH (N=155)** | AFC, Age, BMI | GONAL-f® or Puregon® |
| **Espinós 2017** | **Diet (N=21)** | AFC >13 | GONAL-f® |
|  | **Control (N=20)** | AFC >13 | GONAL-f® |
| **Nyboe Andersen 2017** | **r-hFSH (N=661)** | None | GONAL-f® |
| **Rettenbacher 2015** | **Bemfola (N=220)** | None | Bemfola® |
|  | **GONAL-f (N=113)** | None | GONAL-f® |
| **Strowitzki 2016** | **Ovaleap (N=153)** | None | Ovaleap® |
|  | **GONAL-f (N=146)** | None | GONAL-f® |
| **Devroey 2012** | **r-hFSH (N=375)** | None | Puregon® |
| **Durnerin 2008** | **Control (N=49)** | None | GONAL-f® |
|  | **r-hLH pretreat (N=53)** | None | GONAL-f® |
| **Esteves 2009** | **r-hFSH (N=236)** | Age, Baseline ovarian volume on TVUS, BMI, Serum FSH on Day 2 or 3 of the menstrual cycle, Number of pre-antral follicles between Days 2 and 3 of the menstrual cycle after pituitary down-regulation | GONAL-f® |
| **Devroey 2009** | **r-hFSH (N=750)** | None | Puregon® |
| **Freiesleben 2008** | **r-hFSH (N=159)** | None | Puregon® |
| **Kyrou 2009** | **r-hFSH (N=230)** | None | Puregon® |
| **Lossl 2008** | **Androgen priming (N=53)** | None | GONAL-f® |
|  | **Control (N=50)** | None | GONAL-f® |
| **Nakhuda 2010^‡^** | **(N=104)** | None | GONAL-f® + Repronex® |
| **Nyboe Andersen 2008** | **r-hFSH (N=261)** | Age | GONAL-f® |
|  | **r-hFSH + r-hLH (N=265)** | Age | Luveris® |
| **Requena 2010** | **r-hFSH + HP-hMG (N=46)** | None | Menopur® + GONAL-f® |
|  | **r-hFSH (N=46)** | None | GONAL-f® |
| **Yovich 2012** | **FSH dose <100 IU (N=47)** | AFC, Age, AMH, BMI, Day-2 FSH, Smoking | Puregon® or GONAL-f® |
| AFC, antral follicle count; AMH, anti-Müllerian hormone; BMI, body mass index; hMG, human menopausal gondatrophins; N/A, not applicable to study as different patients had different biomarkers recorded; rFSH, recombinant follicle stimulating hormone; r-hFSH, recombinant-human follicle stimulating hormone; TVUS, transvaginal ultrasound. | | | |
